# RESCUE: a validated Nanopore pipeline to classify bacteria through long-read, 16S-ITS-23S rRNA sequencing

**DOI:** 10.3389/fmicb.2023.1201064

**Published:** 2023-07-20

**Authors:** Joseph R. Petrone, Paula Rios Glusberger, Christian D. George, Patricia L. Milletich, Angelica P. Ahrens, Luiz Fernando Wurdig Roesch, Eric W. Triplett

**Affiliations:** Department of Microbiology and Cell Science, Institute of Food and Agricultural Sciences, University of Florida, Gainesville, FL, United States

**Keywords:** 16S rRNA, bacterial classification, *rrn*, 16S-ITS-23S, microbiome, Nanopore, Illumina, sequencing

## Abstract

Despite the advent of third-generation sequencing technologies, modern bacterial ecology studies still use Illumina to sequence small (~400 bp) hypervariable regions of the 16S rRNA SSU for phylogenetic classification. By sequencing a larger region of the rRNA gene operons, the limitations and biases of sequencing small portions can be removed, allowing for more accurate classification with deeper taxonomic resolution. With Nanopore sequencing now providing raw simplex reads with quality scores above Q20 using the kit 12 chemistry, the ease, cost, and portability of Nanopore play a leading role in performing differential bacterial abundance analysis. Sequencing the near-entire *rrn* operon of bacteria and archaea enables the use of the universally conserved operon holding evolutionary polymorphisms for taxonomic resolution. Here, a reproducible and validated pipeline was developed, RRN-operon Enabled Species-level Classification Using EMU (RESCUE), to facilitate the sequencing of bacterial *rrn* operons and to support import into phyloseq. Benchmarking RESCUE showed that fully processed reads are now parallel or exceed the quality of Sanger, with median quality scores of approximately Q20+, using the R10.4 and Guppy SUP basecalling. The pipeline was validated through two complex mock samples, the use of multiple sample types, with actual Illumina data, and across four databases. RESCUE sequencing is shown to drastically improve classification to the species level for most taxa and resolves erroneous taxa caused by using short reads such as Illumina.

## 1. Introduction

Since the introduction of next-generation DNA sequencing technologies and the first 16S rRNA gene phylogenetic classification publication in 1977 (Fox et al., 1977), molecular use of the 16S rRNA gene has grown and is now implemented in many laboratories and diagnostic clinics (Woo et al., 2008). Although only a 5-year gap exists between the rollout of second-generation technologies, such as Roche 454/Illumina, and third-generation technologies, such as Pacific BioSciences (PacBio) and Oxford Nanopore Technologies (ONT) (Malla et al., 2019), Illumina is preferred for its accuracy. As Illumina amplicon sequencing is FDA-approved, it is also preferred by many diagnostic genomic cores (Collins and Hamburg, 2013; Laehnemann et al., 2016). Hence, the literature is generally limited to the analysis of one or two small hypervariable regions on the 1540 bp 16S rRNA gene, given the constraints of paired-end sequencing length by the Illumina platform (Bukin et al., 2019).

Although individual regions sequenced on Illumina such as V3V4, V4, internal transcribed spacer (ITS), and 23S capture the sequence divergence of many bacterial species (Pei et al., 2009; Snyder et al., 2011; Yarza et al., 2014; Russell et al., 2019; Kui et al., 2021), taxonomic classification and relative abundance calculations can differ greatly based on the hypervariable region sequenced or the primer-set selection, which may misrepresent and also completely omit taxa (Graspeuntner et al., 2018; Darwish et al., 2021). With the recent FDA approval of ONT for clinical diagnostics and an increased interest in a deeper understanding of environmental microbiomes as technologies become more accessible, it is timely that long-read pipelines be established with improved accuracy and confidence in classification compared to short-read sequencing (Yarza et al., 2014; Martínez-Porchas et al., 2016; PRWeb, 2020; De Coster et al., 2021).

The read length constraints of Illumina ultimately limit the accuracy of taxonomic assignment. The invention of single-molecule sequencing such as PacBio and ONT has been the long-read answer, providing reads longer than 300 bp and has been essential for closing genome assemblies (De Maio et al., 2019; Amarasinghe et al., 2020; Petrone et al., 2022). Although ONT was once considered to have inferior quality, their continuously updating chemistry and flowcells are now seeing extremely accurate basecalling in sequencing (Petrone et al., 2022; Sereika et al., 2022, p. 4). Another point of concern in sequencing experiments revolves around the cost per base generated. Some researchers have found approximately $200 per gigabase (Gb) generated for Illumina MiSeq 2 x 250 bp, $1,000 for PacBio RSII, and the listed cost for ONT is ~$750 (Goodwin et al., 2016). In practice, however, researchers can find a reducing cost that is ~$100 per Gb realistically for ONT or less, as owning a $1,000 MinION is achievable and most laboratories are accustomed to sending in samples to a core for Illumina or PacBio due to the drastic difference in machine costs (Ameur et al., 2019; Zhang et al., 2020).

While the near entirety of the bacterial *rrn* operon contains mostly taxonomic diversity, such as 16S rRNA, ITS, and the 23S rRNA (omitting the 5S rRNA) genes, it has only recently become feasible to use long-read sequencing to achieve this genetic capture (Cuscó et al., 2018; Graf et al., 2021; Kinoshita et al., 2021; Kui et al., 2021). Here, we developed and validated a novel *rrn* sequencing pipeline, RESCUE, for robust taxonomic identification across multiple sample types, using the newest Nanopore chemistry (Kit12 Q20+), flow cells (R10.4), and SUP Guppy (v6.1.7) (Oxford Nanopore Technologies Ltd., 2000). To assess the reliability and generalizability of the pipeline, the RESCUE and Illumina MiSeq methodologies are compared in human saliva samples and commercially available mock communities, comparing results obtained with several commonly used taxonomic databases.

In attempts to draw accurate comparisons to previously generated Illumina MiSeq data (V3V4) through amplification of the same DNA aliquots using RESCUE rRNA sequencing, we employed an *in silico* technique in benchmarking to bridge known confounding variables. By bioinformatically extracting the V3V4 hypervariable regions from the RESCUE-generated reads and using four diverse databases, the effect of amplicon length on species-level assignment can be more easily seen as this comparison directly compares how a classification would differ if an identical read was shorted to mirror a V3V4 amplicon.

## 2. Materials and methods

### 2.1. Nanopore experimental design with the R9.4 flow cell

For both trials mentioned later, a detailed graphical layout to understand the sample flow can be better seen (Supplementary Figure 1). For the first experiment, variable *rrn* amplicon length capture was tested across multiple sample types by optimizing PCR extension time. Five polymerase extension times were compared: 1, 4, 8, 16, and 32 min, across six DNA sample types from bulk soil, rhizosphere, apoplastic fluid of *S. lycopersicum*, a Zymo microbial DNA mock community, human saliva, and human feces. Some extension time and sample type combinations are not presented due to a lack of amplification in the saliva and bulk soil samples at 1 min and non-specific binding appearing at approximately 8 min. A total of 20 samples were multiplexed on the R9.4 flow cell.

### 2.2. Nanopore experimental validation design with the flow cell R10.4

To test the application of the *rrn* RESCUE pipeline to accurately classify bacteria, perform in a full-scale experiment, and to compare the results generated by the Illumina platform, four R10.4 (FLO-MIN112) flow cells were loaded with a full 96-plex of barcoded samples on each (Ahrens et al., 2022). Twenty-one saliva samples had also been previously sequenced on the Illumina MiSeq (2 x 300 bp) platform (Ahrens et al., 2022). Two additional saliva samples were provided by a collaborator at the University of Florida's College of Dentistry (P65 and P95), amplified at 1 and 4 min. A ZymoBIOMICS Microbial Community DNA Standard (Cat. No. D6305) was included on each run as well (“Mock,” *N* = 4). On the fourth flow cell, four biological replicate extractions of the ZymoBIOMICS Gut Microbiome Standard (Cat. No. D6331) were included to test a known gut community (“Gut,” *N* = 4). All samples were amplified at 4-min extension times using 10 ng of input DNA.

### 2.3. PCR design, quantification, and purification

**Figure 1:**
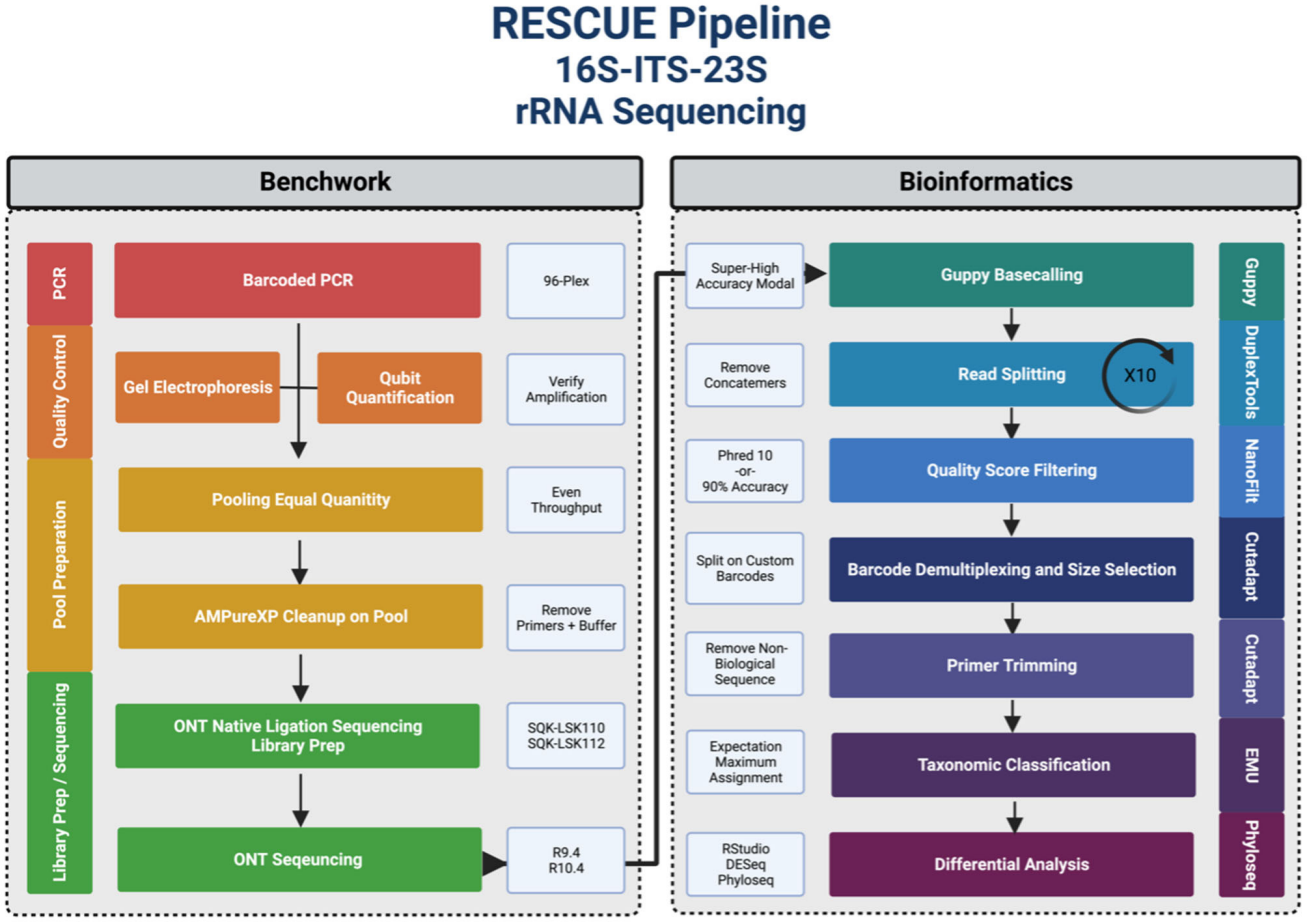
Graphical abstract of the RESCUE, rRNA operon Nanopore sequencing pipeline.

A graphical abstract of the steps and softwares leveraged by RESCUE can be summarized in (Figure 1). Primers used in the *rrn* reactions were 27F (5'-AGRRTTYGATYHTDGYTYAG-3') and U2428R (5'-CCRAMCTGTCTCACGACG-3') (Graf et al., 2021). A more detailed analysis of methods is published on GitHub https://github.com/josephpetrone/RESCUE/blob/master/detailed_steps.md (Petroovius, 2023b). Custom barcodes were adapted from PacBio 16S barcodes (Procedure Checklist., 2021). 96-well plates were multiplexed with eight forward primers and twelve reverse primers. Phusion Hot Start II High-Fidelity polymerase (cat no. F549L, Thermo Fisher Scientific, Carlsbad CA) and 10 ng of source DNA were used in each amplification. While standardized protocols such as the “earth microbiome project” call for a low-fidelity Taq polymerase, we chose to employ a high-fidelity polymerase to mitigate any additional amplification errors. Sample DNA extraction was performed as described previously (Russell et al., 2019; Ahrens et al., 2022). Thermocycling conditions included the following: 98°C for 30 s initially, 30 cycles of 98°C for 10 sec, 71.5°C for 30 sec, 72°C for “N min,” followed by a final extension at 72°C for 7.5 min carried out on an Eppendorf nexus GX2 mastercycler (Eppendorf AG, Hamburg, Germany). “N min” used includes 1, 4, and 8 min. To carry out sequencing on the R10.4 flow cell, 4 min was exclusively used.

PCR products were quantified directly using Qubit 2.0 fluorometer and HS 1X buffer (Thermo Fisher Scientific, Waltham, MA, USA) to ensure even throughput in the DNA pool. A minimum of 3 μg of pooled DNA was used for multiple library constructions. AMPure XP beads (Beckman Coulter Inc.) were added at 0.6X v/v concentration for purification, and the elution was carried out in 100 μL of EDTA-free elution buffer.

### 2.4. Library preparation and sequencing

A 1:10 dilution of the amplicon pool was quantified using Qubit 1X dsDNA HS kit, and 1.5 μg was aliquoted and brought to 50 μL with sterile nuclease-free water. For the R9.4 experiment, the DNA was processed using ONT ligation sequencing kit-10 (SQK-LSK110) and loaded onto an R9.4 FLO-MIN106D flow cell. For the R10.4 trials, the newest ONT ligation sequencing kit-12 (SQK-LSK112) was used, and the library was loaded onto four R10.4 FLO-MIN112 flow cells. To avoid wasting throughput on sheared amplicons, vortexing was replaced with gentle flicking, and wide-bore tips were used for DNA handling. Libraries were sequenced for 72 h on the GridION Mk1. The R9.4 and R10.4 flow cells were sequenced through MinKNOW v21.11.6 and MinKNOW v21.11.7, respectively.

### 2.5. Read processing and classification

All R10.4 data were first re-basecalled using Guppy v6.1.5 on the super high accuracy basecalling modal “SUP.” Basecalled reads were then ran through the RESCUE pipeline at this stage, available at https://github.com/josephpetrone/RESCUE (Petroovius, 2023a). The reads were run through Duplex Tools v0.2.9 (Duplex Tools., 2022) to split concatenated reads by Nanopore adapter ten times. Single fastq reads were observed to be made up of continual sequences “concatenated” together due to quick succession through the pore and consisted of entire 4.5 kb reads digitally stitched together. Ten rounds were needed despite the “allow-multiple” option being selected as each round produced additional splitting. This step has been fixed in the newest Guppy release (v6.1.7) with “—do_read_splitting” enabled. The split reads were then re-filtered by average Phred ≥ 10 using NanoFilt v2.7.1 (De Coster et al., 2018). Cutadapt v3.4 (Martin, 2011) was run to demultiplex samples, with the requirement that all 16 bases of each dual barcode align with zero mismatches and the barcodes be found in the correct orientation, such that all negative strands were reverse complemented. The demultiplexing stage also utilizes a minimum and maximum, trimmed-read cutoff of 3 kb and 7 kb, respectively. Cutadapt was then used again to remove all non-biological nucleotides including DNA primers. NanoPlot v1.30.1 (De Coster et al., 2018) was used for descriptive statistics on the runs.

The reads were then classified using the EMU v3.4.1 (Curry et al., 2021, 2022) program by mapping in four published and verified databases: an *rrn* operon database, ncbi_202006 “NCBI RRN” (Kinoshita et al., 2021), and three full-length 16S databases, Silva-138, EMU, and Ribosomal Database Project “RDP” (McMurdie and Holmes, 2013; RStudio Team, 2020). The main database of RESCUE, the NCBI RRN, was made by Kinoshita et al. (2021) by pulling rrn operon DNA sequences from NCBI RefSeq and reviewing GenBank entries. The database was formatted for EMU here, and the taxids were updated to the current denotations. The RESCUE pipeline contains command-line flags to choose one of the four databases mentioned, any database hosted by the EMU server, and a self-created database with a -d flag in the RESCUE package that points to a database folder. The phyloseq object generated includes classified read counts, the taxonomy of the database hits, and a personalized mapping file, which was modeled after the Qiime format (Caporaso et al., 2010).

### 2.6. Illumina MiSeq sequencing and RESCUE comparisons

For the Illumina sequencing comparisons against the Nanopore data, the trimmed and merged fastq output from Illumina MiSeq V3-V4 (2 X 300 bp), described as previously published (Russell et al., 2019; Ahrens et al., 2022), was used in the analysis. The original DNA aliquot used for sequencing the Illumina datasets was also used with the described RESCUE primers to amplify the *rrn* operon and sequence with Nanopore. The raw fastq files for each MiSeq sample (one fecal and one saliva for R9.4; 21 saliva samples in total for R10.4) were run through the EMU classifier and four mentioned databases. The default compositional values obtained for the Zymo mock communities were found through the online manual for the theoretical 16S rRNA abundances (ZYMO Research, 2022).

### 2.7. Pseudo-V3V4 generation

The hypervariable V3V4 regions were extracted and trimmed from the *rrn* reads *in silico* using Cutadapt v3.4 (Martin, 2011) to mimic the exact primer binding sites of the MiSeq V3V4 primers previously used. The extracted V3V4 reads were then classified through the EMU program using the four databases. The Pseudo-V3V4 reads serve to definitively show that shortening the size of an amplicon has negative impacts at species-level classifications. These reads also serve as an important comparison to the true Illumina data, as any differences between them show differences that a researcher might expect when varying library prep and sequencing platforms.

### 2.8. Phyloseq handling and NMDS creation

Phyloseq v1.38.0 was used to produce a phyloseq object from the EMU output files. To generate the phyloseq object from four separate databases at both the species and genus levels, first, the RESCUE output csv was read through the provided RScript and converted into a large data frame with taxa as rows and sample names as columns to create an OTU table. The OTU tables were then aggregated on a dataframe that only included both the species and genus columns. This was done to remove results that did not have classification at the genus level and to standardize the nomenclature of the taxa before assigning OTUs. Cases where the species-level taxonomy was blank were given an “Unclassified” designation before aggregating. Samples were rarefied to 1,900 reads and filtered to remove taxa that were not present at >5 reads in 3 samples. To generate the NMDS plots, the “ordinate” package was used with the grouping column set to “read type,” “Bray,” or “binomial” as the method, and a max of 1,000 permutations were allowed before reaching the best RMSE solution twice. The ordination data was then graphed with ggplot2.

## 3. Results

### 3.1. Nanopore R9.4 trial run metrics and read recovery

The first multiplexed library was run on an R9.4 flow cell until exhaustion (~72 h) and base-called with Guppy v5.1.12 (SUP). A detailed graphical flowchart of the sample design can be found (Supplementary Figure 1). A total of 1.344 x 10^6^ reads ≥ Phred Q10 (5.627 Gb) were generated, with a mean quality score of 13.1 and a mean sequence length of 4,578 nucleotides (Table 1). Small peaks were visible in the raw read histogram of the MinKNOW GUI at 10 kb, 15 kb, and 20 kb, which corresponded to the incorrect stitching of multiple unique strands passing through a pore in quick succession. These reads are referred to as concatenated reads as they are artificially stitched together during the ONT raw file generations. Two rounds of read splitting on internal adapters recovered an additional 15,079 reads for a total of 1.359 x 10^6^ reads (Table 1). Demultiplexing this 20-plex run with the first, barcode mismatch-tolerable iteration of the demultiplexer attributed 9.38 x 10^5^ reads to a correct barcode pair, with an average of 4.66 x 10^4^ reads/sample, ranging from 1.21 x 10^4^ to 9.08 x 10^4^ reads/sample. As 76 barcodes were unused on the flowcell, we had the ability to attempt demultiplexing as if 96 samples were run. This uncovered that 0.0059% of the reads incorrectly demultiplexed into these unused barcodes. The per-sample relative abundance threshold was then set to 0.0059%, to account for the percentage of reads demultiplexed into the unused barcode combinations across the R9.4 run. This threshold was used to remove the possibility of taxa only being present due to the slight chance of improper demultiplexing by using reads in the null controls as an internal validation mechanism. Over 70% of the reads post-trimming retained an average Phred score above Q12 (93.7% raw read accuracy).

**Table 1:** ONT R9.4 read quantity, quality, and length statistics for the R9.4 Kit 10 run.

**R9.4 Run**	**Step**	**Reads (•10^6^)**	**Bases (Gb)**	**Avg Len**	**Med Len**	**Avg Q-score**	**Med Q-score**	**% ≥Q12**	**% ≥Q15**
Run1	1-Basecalling	1.34	5.63	4,186	4,578	13.1	12.9	67	17
	2-Read Splitting	1.36	5.63	4,140	4,571	13.1	12.9	67	17
	3-Demultiplexing	0.996	4.47	4,491	4,625	13.2	13.1	69	19
	4-Trimming	0.938	4.22	4,498	4,521	13.3	13.2	70	20

Each step listed along the pipeline can be seen in each row. Starting at the demultiplexing step, all unclassified or not demultiplexed data was removed from the statistics.

### 3.2. Increasing polymerase extension time to detect unlinked-*rrn* taxa

**Figure 2:**
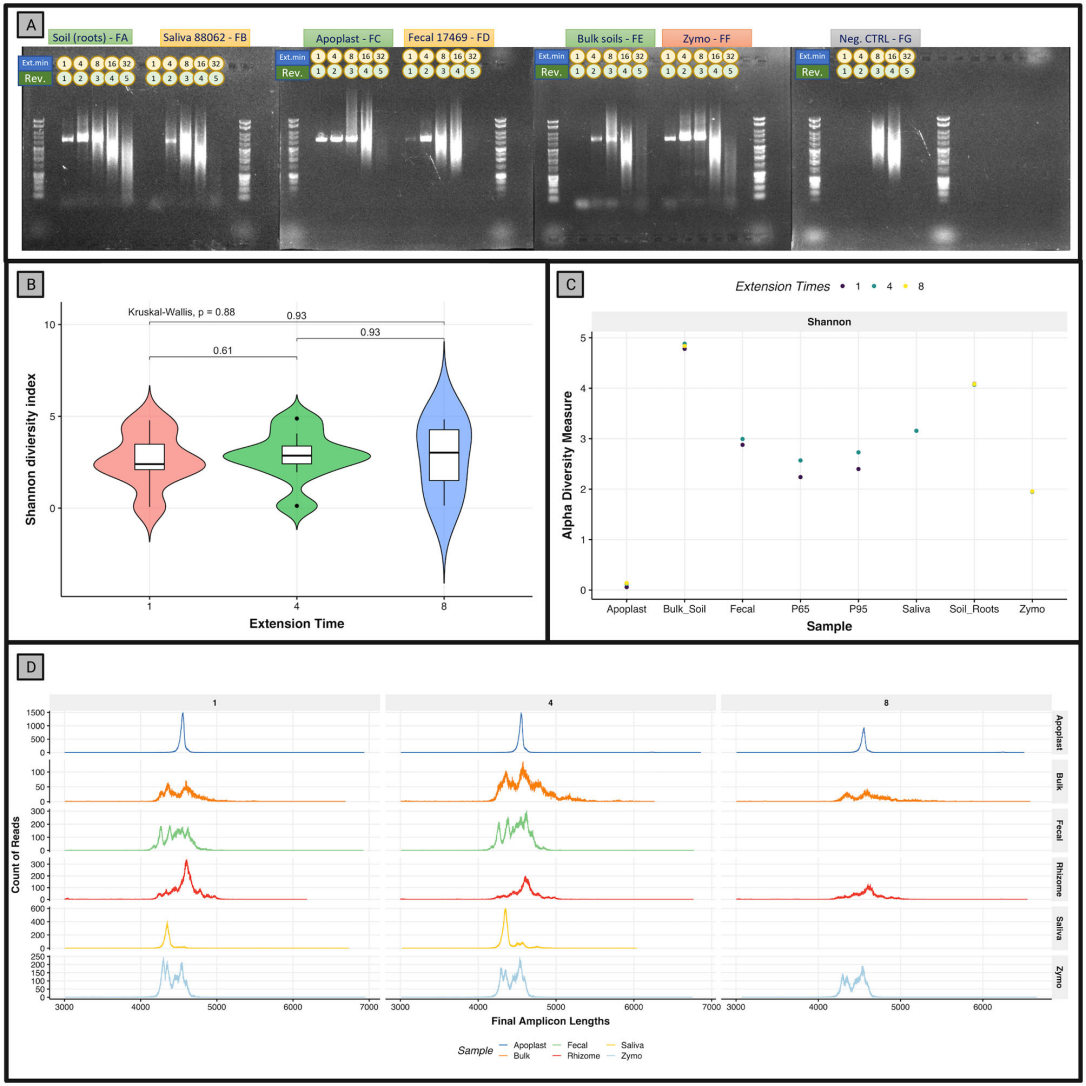
Agarose gel of the library and alpha diversity of the r9.4 analysis. **(A)** Gel electrophoresis (1% agarose) results for the R9.4 trial by sample type and extension time. Sample type is shown above each section of the gel while the “Ext_min” row refers to the PCR extension time used. A total of 4 μL of PCR product was mixed with 1 μL of 6x Tri-Track loading dye before loading 4 μL into the gel. Gels were run for 30 min at 100 V and shown against 1 μL of a Promega Benchtop 1 Kb Ladder. Reverse barcode numbers (Rev) are depicted. **(B)** Alpha diversity measures of Shannon diversity plotted across all extension times tested. Samples are rarefied to 12,171 reads/sample. **(C)** Species richness is plotted for each rarefied sample on the x-axis while the Shannon diversity index is plotted on the y-axis. Extension time variables can be seen as the color of the data point. **(D)** Read lengths of demultiplexed, untrimmed reads by extension time and sample type.

Bacterial genomes with 64-128 Kb of intergenic space between the 16S and 23S rRNA genes could theoretically be detected at an extension time of 8 min, with the extension time of Phusion^®^ High-Fidelity Hot Start Polymerase at 15–30 s per kilobase. It is important to note that a long amplification polymerase may be better suited for capturing longer *rrn* amplicons, but a tradeoff with accuracy is to be expected. Although significant smearing was observed in the *rrn* PCR products (Figure 2A), the N50 observed from the sequencing output was approximately 4.2 Kb (Table 1). In the final trimmed reads, the average and median read lengths converged at approximately 4.5 Kb.

Distinct peaks in read lengths across the R9.4 trial appeared unique to the sample type and their respective taxa, almost as a visual phylogenetic fingerprint (Figure 2D). Extension time appeared to only affect the quantity of DNA produced in the PCR process, as opposed to increasing the sequence length, as the average DNA concentration increased across the extension times, 1, 4, and 8 min (4.9, 11.6, and 16.6 ng/μL, respectively). When separated by sample type, overall alpha diversity does not change significantly (Kruskal–Wallis: *p* = 0.88), (Figure 2B). Optimal extension time and the resulting microbial diversity may vary by sample type, as was observed in the saliva samples P65 and P95, for which alpha diversity increased with extension time (1–4 min). A 4-min extension time produced the most amplicon product, with less smearing than was observed at higher extension times.

### 3.3. R9.4 *rrn* analysis can accurately classify species in the mock community

**Figure 3:**
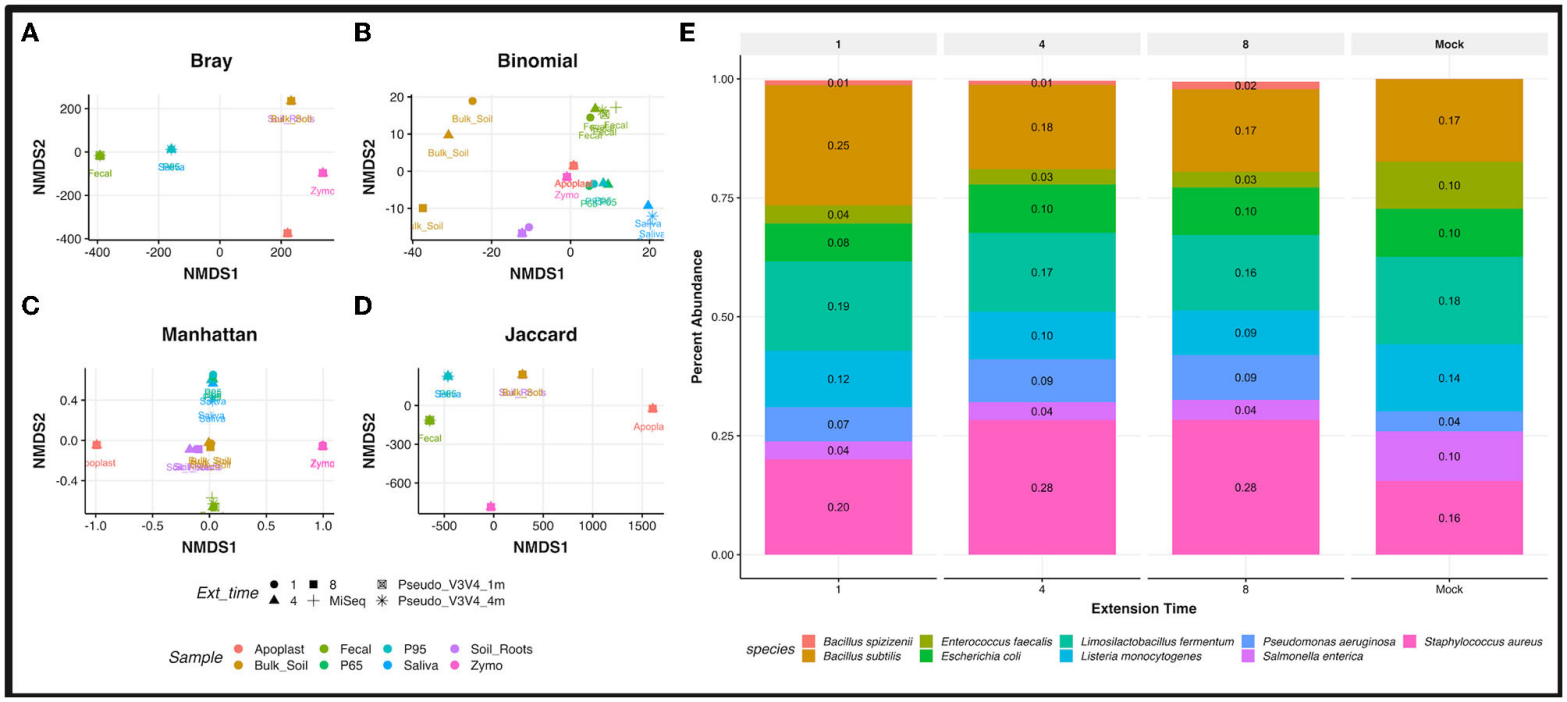
Sample type and trial comparisons. Non-metric multidimensional scaling (NMDS) ordination plot using “Bray” **(A)**, “binomial” **(B)**, “Manhattan” **(C)**, and “Jaccard” **(D)** distance matrix. Calculations metrics from the “ordinate” function of the Phyloseq package. All samples were rarefied to the lowest throughput of 12,171 reads/samples, transformed to relative abundance at the species level, and thresholded to a minimum of 0.0059 relative abundance before plotting. Samples are grouped by color while extension time variables are shown by the data point's shape. **(E)** Relative abundances observed in the Zymo Community DNA standard across PCR extension times are plotted for the R9.4 trial against the theoretical (expected) abundances from the manufacturer (Mock). All samples are rarified.

As expected, the lowest species richness in the R9.4 trial was observed in the commercial Zymo microbial DNA standard (mock) samples, designed to include only eight taxa (Figure 2C). After rarefaction at 1,900 reads and abundance filtering for barcode crosstalk, taxonomic classification, and relative abundances were comparable to the mock community composition and abundances listed by the manufacturer (Figure 3E). All eight species were resolved at the species level in the *rrn* samples. An additional classification of *Bacillus spizizenii*, a subspecies of *Bacillus subtilis*, was identified with the lowest relative abundance average of 1.1% (Figure 3E). The relative abundances of *Staphylococcus aureus* appeared slightly over-represented by *rrn* sequencing, while *Enterococcus faecalis* was slightly under-represented when compared to the expected theoretical values of the mock. However, this variation does not appear to be in response to extension times.

Non-metric multidimensional scaling (NMDS) demonstrated distinct clustering of *rrn* samples by sample type on the rarefied and filtered dataset. Distinct clusters were observed across all sample types, with extension times tightly clustering (Figures 3A–D). Separation based on the sample host, such as a human or plant, was also observed in several distance matrices (Bray, Binomial, Manhattan, and Jaccard). The Illumina MiSeq V3V4 data for the fecal and saliva sample clustered alongside the Pseudo-V3V4 reads in their respective *rrn* sample type clusters (Figures 3A–D).

### 3.4. R10.4 and Kit12 chemistry significantly increase read accuracy

Four fully multiplexed libraries, each with 96 samples, were run on the newer R10.4 flow cells and prepared using the most current Kit12 Q20+ ligation sequencing kit (SQK-LSK112). An average of 11.46 Gb of data (≥ Q10) were generated on each flow cell after basecalling with Guppy v6.1.7 SUP. Albeit an initial Q-score cutoff of Phred-10, the four runs averaged having over 70% of the reads >Q15. Initially, the first run was basecalled on Guppy v5.1.13, with a mean Q-score of 16.3. After re-basecalling with the newest Guppy (v6.1.7), the average Q-score increased by almost a full Phred point, 17.3. Reads were split ten times to ensure no concatenated reads remained. This resulted in the splitting and recovery of 9.0 x 10^5^ additional reads on average, with run 2 recovering about 1.35 x 10^6^ reads (Table 2). Re-filtering the split reads by average Q-score resulted in a median read length of 4,450 nucleotides with an average Q-score of 17.8.

**Table 2:** Read quantity, quality, and length statistics for the R10.4 Kit 12 runs.

**R10.4 Run**	**Step**	**Reads (•10^6^)**	**Bases (Gb)**	**Avg Len**	**Med Len**	**Avg Q-score**	**Med Q-score**	**% ≥Q12**	**% ≥Q15**
Run1	1-Basecalling^*^	2.32	9.78	4,207	4,447	16.3	16.6	89	66
	1-Basecalling	1.85	9.99	5,406	4,480	17.1	17.3	94	73
	2-Read Splitting	2.99	9.95	3,331	4,395	17.6	17.9	93	75
	3-Filtering	2.92	9.72	3,333	4,406	17.8	18.0	95	77
	4-Demultiplexing	0.995	4.41	4,660	4,615	19.3	19.6	99	91
	5-Trimming	0.995	4.25	4,490	4,502	19.9	20.2	99	92
Run2	1-Basecalling	2.29	12.65	5,517	4,488	17.2	17.4	92	72
	2-Read Splitting	3.64	12.60	3,456	4,428	17.6	17.9	91	74
	3-Filtering	3.55	12.25	3,452	4,432	17.9	18.1	94	76
	4-Demultiplexing	1.19	5.52	4,656	4,608	19.4	19.7	99	90
	5-Trimming	1.19	5.33	4,494	4,489	20.0	20.3	99	92
Run3	1-Basecalling	2.19	12.67	5,772	4,634	16.9	17.1	89	68
	2-Read Splitting	3.24	12.62	3,895	4,466	17.4	17.8	89	71
	3-Filtering	3.15	12.33	3,906	4,469	17.6	17.9	91	73
	4-Demultiplexing	1.19	5.51	4,650	4,604	19.5	19.9	98	91
	5-Trimming	1.19	5.33	4,496	4,483	20.1	20.5	98	92
Run4	1-Basecalling	2.52	10.54	4,185	4,481	16.9	17.1	89	68
	2-Read Splitting	2.60	10.53	4,056	4,479	16.9	17.1	89	68
	3-Filtering	2.58	10.46	4,058	4,480	17.0	17.2	90	69
	4-Demultiplexing	1.00	4.64	4,629	4,534	18.6	18.9	98	87
	5-Trimming	1.00	4.48	4,470	4,401	19.3	19.5	98	88

For the demultiplexing and trimming steps, statistics exclude any unclassified reads as well as demultiplexed data. NanoStat was used on raw fastq files to generate the output. For Run1, the initial basecalling was also performed with Guppy v5.1.12, denoted by “^*^”. Avg, average; Med, median.

Demultiplexing constraints on the R10.4 were stringent, ensuring the quality of the alignments over the quantity of reads remaining. No mismatches were allowed in either dual-indexed barcode, and a read length between 3 and 7 kb was required. After demultiplexing, an average of 1.09 x 10^6^ reads per flow cell remained, a 36% retention from the pipeline's previous filtering step (an average of 3.05 x 10^6^ reads). Altering mismatch parameters is explored further in Supplementary Table 1. Non-biological nucleotides were removed, resulting in an increase in the median quality scores to approximately Q20.1 with 91% of the total reads being ≥ Q15 and an average read length of 4,488 ± 26 bases (Table 2). A per-sample relative abundance of 0.0073% was used to remove the possibility of barcode crosstalk based on the percentage of reads in the null control barcode.

### 3.5. RRN RESCUE enables deeper classification with superior evenness compared to short-read methodologies

**Figure 4:**
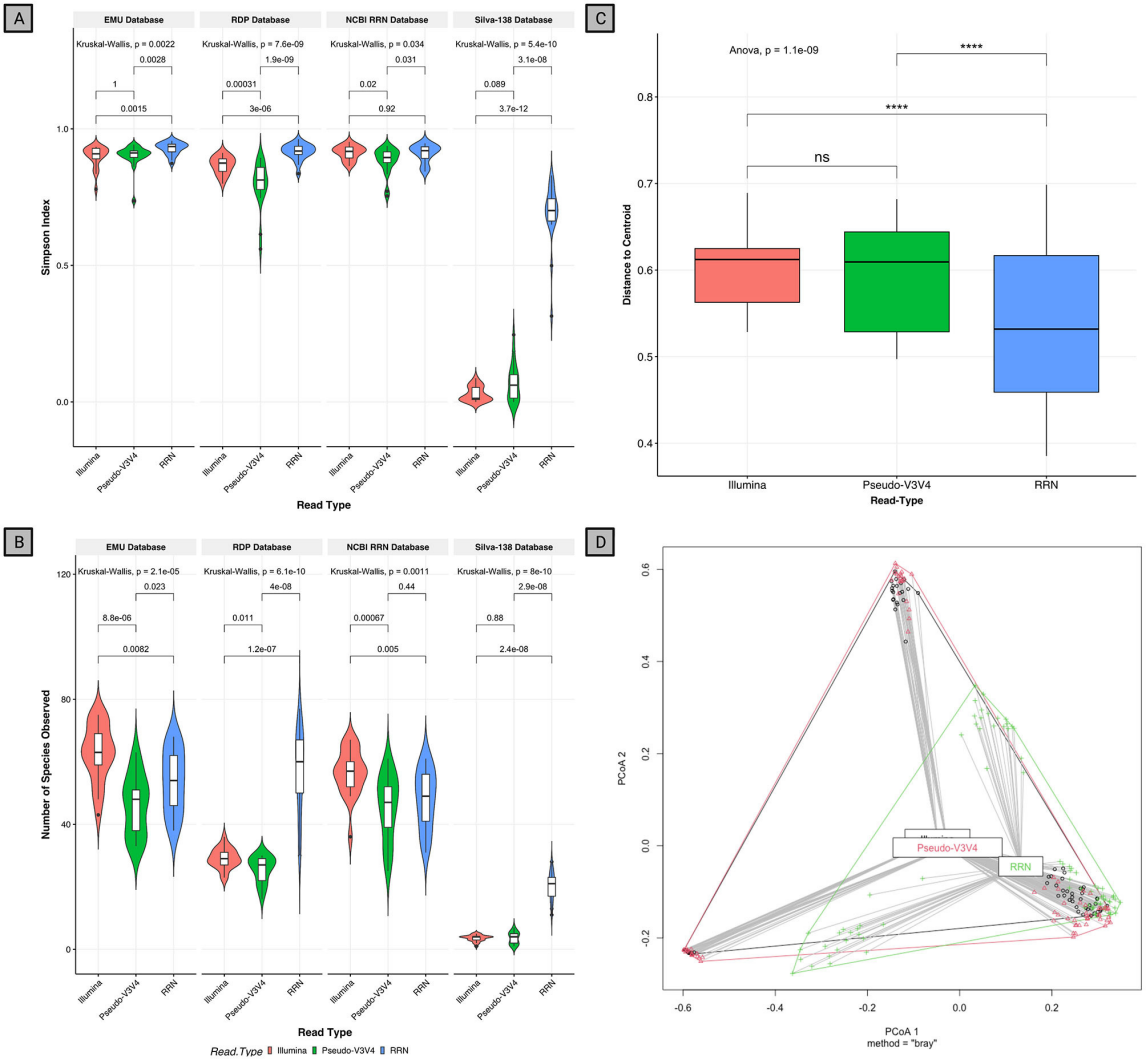
Alpha- and beta-diversity measures of the R10.4 rrn Illumina comparison. **(A)** Alpha diversity by the measure of Simpson Evenness and **(B)** Observed richness. The database is faceted on top of each read grouping by database while the sequence type can be seen as red (Illumina), green (Pseudo-V3V4), and blue (RRN). Kruskal–Wallis and pairwise chi-squared were done and p.adj is shown. **(C)** Beta-dispersion metrics of distance to the centroid. Sprawl between sample centers can be seen on the y-axis while each sequence type is plotted separately on the x-axis. Non-parametric ANOVA and pairwise *t*-test are shown here. **(D)** Beta-dispersion PCoA by Bray–Curtis distance matrix. Each sequence type can be seen as a distinct shape or by color: Illumina (black), Pseudo-V3V4 (red), and RRN (green).

To test our hypothesis that short reads can negatively impact classification, irrespective of the sequencing platform used, four rRNA gene datasets (EMU, RDP, Silva-138, and NCBI RRN) were used to classify the same saliva DNA sources (*n* = 21) from Illumina V3V4, 16S-ITS-23S ONT (*rrn*), and Pseudo-V3V4. The Pseudo-V3V4 reads were extracted from the full *rrn* reads *in silico* to artificially mimic the size of a V3V4 amplicon while still being generated from the RESCUE *rrn* primers. In this analysis, differences between the Pseudo-V3V4 reads and the *rrn* reads can be attributed to read length while differences in the Illumina V3V4 data can be attributed to primer bias, library prep, and sequencing platform biases. Communities found by *rrn* sequencing were significantly more diverse at the species level than those from the two short-read sets (Illumina and Pseudo-V3V4) across almost all the databases (Simpson's index at species level, Kruskal–Wallis: *p* = 0.0022, *p* = 7.6 x 10^−9^, *p* = 0.034, *p* = 5.4 x 10^−10^; Emu, RDP, NCBI RRN, and Silva-138, respectively) (Figure 4A). The *rrn* reads were significantly more diverse compared to the Illumina and Pseudo-V3V4 data across all databases (Simpson's index at species level, Wilcox: [*p* = 0.0015 and *p* = 0.0028], [*p* = 3.0 x 10^−6^ and *p* = 1.9 x 10^−9^], [ *p* = 0.92 and *p* = 0.031], [*p* = 3.7 x 10^−12^ and *p* = 3.9 x 10^−8^]; [*rrn* vs. Illumina & *rrn* vs. Pseudo-V3V4] Emu, RDP, NCBI RRN, and Silva-138, respectively).

RRN reads also contained a greater number of unique species than Pseudo-V3V4 reads in all databases except the NCBI RRN database (observed richness, Wilcox: *p* = 0.023, *p* = 4.0 x 10^−8^, *p* = 0.44, *p* = 2.9 x 10^−8;^ EMU, RDP, NCBI RRN, and Silva-138, respectively) (Figure 4B). More taxa were observed in Illumina V3V4 reads as compared to *rrn* reads through the EMU and NCBI RRN database despite the lower Simpson diversity (Wilcox: *p* = 0.0082, *p* = 0.005).

Between the samples and within each read type, a pattern emerges of the variability among classifications when using short reads across all four databases. The beta-dispersion distance between samples of *rrn* reads is significantly less than both Illumina and Pseudo-V3V4 reads (ANOVA: *p* = 1.1 x 10^−9^; *t*-test: *p* < 0.0001, *p* < 0.0001; Illumina and Pseudo-V3V4, respectively), while any difference between Illumina and Pseudo-V3V4 reads are negligible (Figures 4C, D). The *rrn* RESCUE community clustered more tightly compared to those of Illumina and Pseudo-V3V4. Stratifying beta-dispersion to show differences between databases and not just read type allows the ability to detect database inherent biases. At the species level, the SILVA-138 database produced classifications that were significantly closer to the centroid than to all other databases (Wilcox: *p* = <0.001) (Supplementary Figure 3C). At the genus level, the database classifications were much more even and Silva-138 produced genera classification without significant differences in beta-dispersion compared to the other databases (Supplementary Figure 3D).

These significant findings interestingly seem to reverse when looking at the same metrics but at the genus level (Supplementary Figures 3A, B). No significant differences were found between the *rrn* and Pseudo-V3V4 reads for the Simpson index, and only one significant difference was observed between the *rrn* and Pseudo-V3V4 reads when using the RDP database (Wilcox: *p* = 0.0013). As for the Illumina reads at the genus level, the samples were consistently more significantly diverse and contained more unique genera than Pseudo-V3V4 and *rrn* data for all databases except for the RDP database. This may show how genus-level analysis does not significantly differ but increasing read size to the entire *rrn* operon enables species-level classification.

### 3.6. R10.4 improves species-level confidence in mock samples

To determine whether RESCUE sequencing with the ONT R10.4 kit-12 chemistry was more effective in species-level detection of mock bacterial communities, four replicates of both the ZymoBIOMICS Microbial DNA Standard and ZymoBIOMICS Gut Microbiome were included across the four runs. This analysis is entirely on RESCUE *rrn* reads and does not use any short-read comparison. First, true error rates of RESCUE reads were analyzed by aligning all classified reads from the Zymo samples against their representative genomes available on the manufacturer's website. Despite retaining a higher error rate at the beginning of the reads aligning to the Zymo mock provided genomes, the error rates toward the middle of the 4.5 kb *rrn* gene sequence allow for ~99% accuracy toward the main portion of the hypervariable region that allows for species-level classification (Supplementary Figure 2).

**Figure 5:**
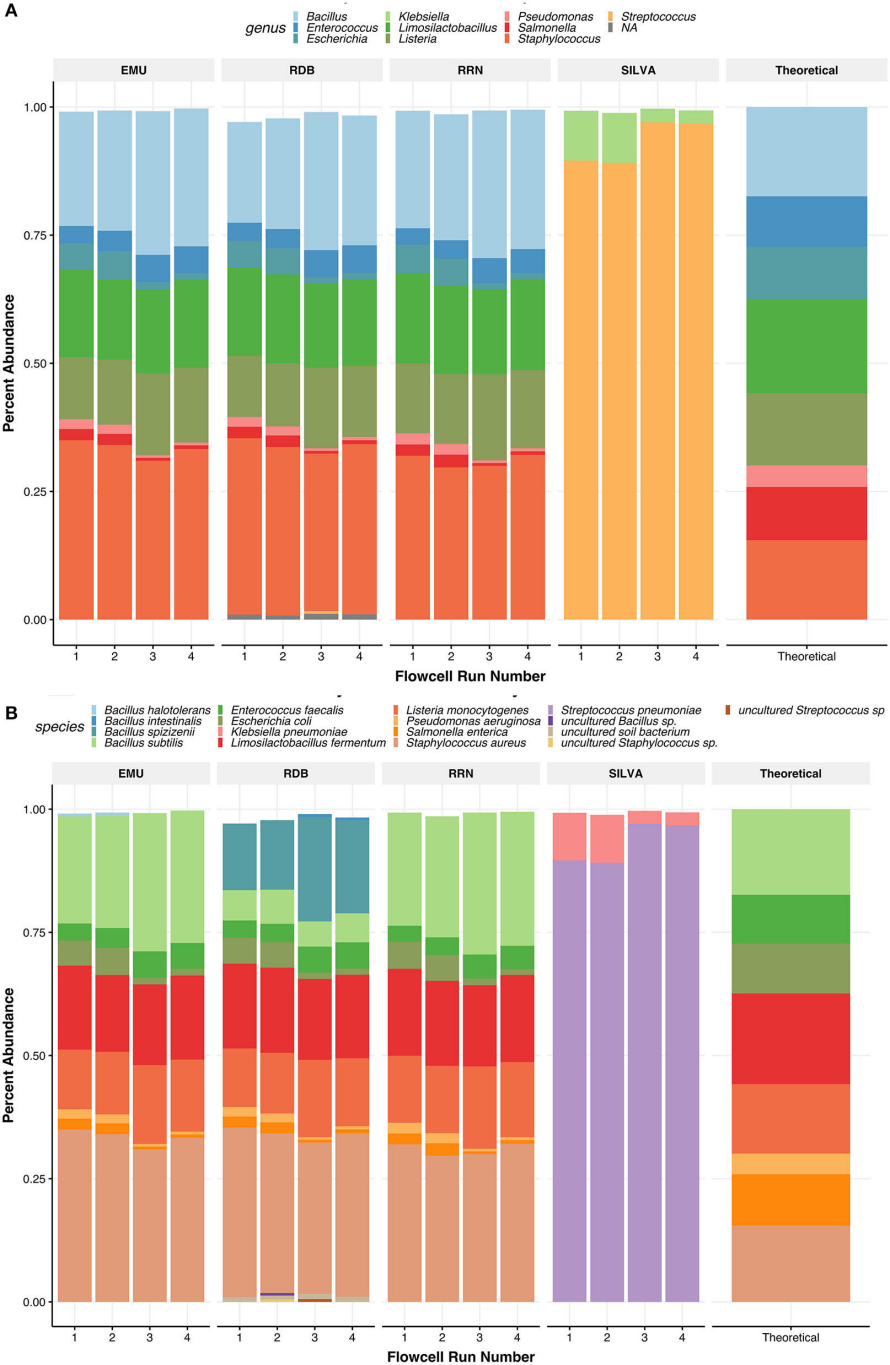
Relative abundances of taxa found in the ZymoBIOMICS Microbial DNA Standard communities. Panels show relative abundance at the genus level **(A)** and the species level **(B)**. Taxa are colored by species or genus found in the legend of each plot. The values for the relative abundance of the mock were pulled from the website of the supplier. For species level, relative abundances can be more accurately seen as their abundance in each bar graph to 2 digits. Each barplot on the x-axis represents the replicate number of the Zymo samples used.

**Figure 6:**
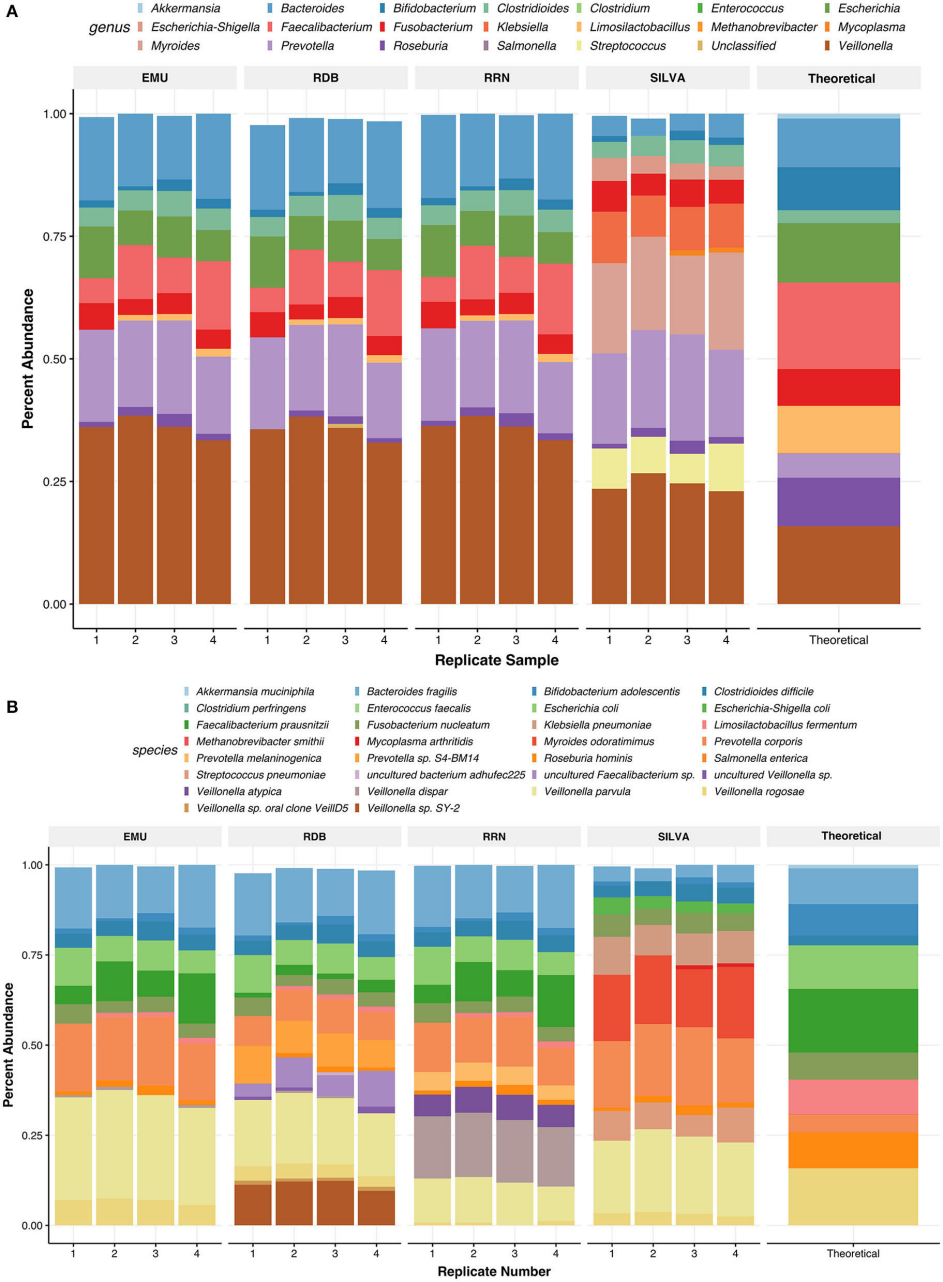
Relative abundances of taxa found in the ZymoBIOMICS Gut Microbiome Standard communities. Panels show relative abundance at the genus level **(A)** and the species level **(B)**. Taxa are colored by species or genus found in the legend of each plot. The values for the relative abundance of the mock were pulled from the website of the supplier. For species level, relative abundances can be more accurately seen as their abundance in each bar graph to 2 digits. Each barplot on the x-axis represents the replicate number of the Zymo samples used.

RESCUE sequencing could accurately resolve the correct composition down to the species level for all eight bacterial taxa of the first mock community using any of the four classification databases (Table 3; Figures 5B, 6B). Using the EMU database led to the identification of a small percentage of *Bacillus halotolerans* (0.29%, Figure 5B), but this disappeared after per-sample taxa filtering at the rate of barcode false attribution observed in the dataset (0.0073 relative abundance, Table 3). Moreover, using the RDP dataset led to the identification of a substantial portion of *Bacillus subtilis* as one of two subspecies, *Bacillus spizizenii* (16.9 %) or Bacillus *intestinalis* (0.30 %), and the identification of an additional uncultured soil bacterium (0.98%) (Table 3). The relative abundances across four databases (NCBI RRN, EMU, Silva-138, and RDP) were comparable, except for the subspecies classification of *Bacillus spizizenii* and *Bacillus subtilis* using the RDP. As for zymo mock classifications using the Silva-138 database, strikingly only *Klebsiella pneumoniae* (6.17%) and *Streptococcus pneumoniae* (93.22%) were detected after abundance thresholding. This strange occurrence would not be resolved if looking at the genus level. Within samples and across four runs, the batch effects of RESCUE with the mock sample were low, with the highest standard deviation among replicates being approximately 1.5% (Figures 5A, B). At the genus level across all four databases, any incomplete taxa missing genus-level classification are completely removed from the datasets (Figure 5A). Among databases, the genus level classification was nearly identical for each sample except for the Silva-138 samples; however, relative abundances did vary, e.g., *Bacillus* was twice the anticipated abundance (observed average = 25.10%; theoretical = 17.40%).

**Table 3:** ONT R10.4 output classification of the 4.5 kb *rrn* reads within the ZymoBIOMICS microbial DNA standard.

**Genus**	**Species**	**NCBI RRN**	**EMU**	**RDP**	**Silva-138**	**Theoretical**
	*Uncultured soil bacterium*			0.98		
*Bacillus*	*Bacillus halotolerans*		0.29			
*Bacillus*	*Bacillus intestinalis*			0.30		
*Bacillus*	*Bacillus spizizenii*			16.92		
*Bacillus*	*Bacillus subtilis*	25.89	24.88	6.02		17.40
*Bacillus*	*Uncultured Bacillus sp*.			0.13		
*Enterococcus*	*Enterococcus faecalis*	4.10	4.50	4.47		9.90
*Escherichia*	*Escherichia coli*	3.33	3.34	3.23		10.10
*Klebsiella*	*Klebsiella pneumoniae*				6.17	
*Limosilactobacillus*	*Limosilactobacillus fermentum*	17.25	16.54	16.97		18.40
*Listeria*	*Listeria monocytogenes*	14.81	13.85	13.43		14.10
*Pseudomonas*	*Pseudomonas aeruginosa*	1.35	1.20	1.22		4.20
*Salmonella*	*Salmonella enterica*	1.47	1.41	1.44		10.40
*Staphylococcus*	*Staphylococcus aureus*	30.95	33.33	32.67		15.50
*Staphylococcus*	*uncultured Staphylococcus sp*.			0.13		
*Streptococcus*	*Streptococcus pneumoniae*				93.11	
*Streptococcus*	*uncultured Streptococcus sp*.			0.14		

Relative abundances are shown as the average across four replicates after applying sample abundance threshold filtering. Four databases were tested: the NCBI RRN database (NCBI RRN), the Ribosomal Database Project (RDP), the EMU database (EMU), and the Silva-138 database (Silva-138). The values for the relative abundance of the Mock were obtained through the supplier's website and attached documents. Taxa that were not found using specific databases are presented as missing values.

Using RESCUE, similar species-level diversity of the second mock, the gut mock community, was observed across all three read types, except for *Akkermansia muciniphila*, which fell below the limit of detection ~0.97% (Figures 6A, B). For two theoretical taxa, *Veillonella rogosae* and *Prevotella corporis*, there were misclassifications among other species of the genera across all four databases (Table 4). *Veillonella rogosae* was the only listed *Veillonella* bacterium included in the mock culture at an abundance of 15.87%, and all four databases were able to classify varying amounts (0.69, 3.72, and 6.79, 3.18%; NCBI RRN, RDP, EMU, Silva, respectively). Yet, all four databases primarily classify the bacterium as *Veillonella parvula* (11.56, 18.41, 28.63, 21.27%; NCBI RRN, RDP, EMU, Silva, respectively). Three databases also picked up *Veillonella dispar* except for Silva-138 (17.25, 0.33, 0.59%; NCBI RRN, RDP, EMU, respectively). NCBI RRN also uniquely detected *Veillonella atypica* while RDP also classified *Veillonella* sp. SY-2 (11.33%), *Veillonella sp. oral clone VeillD5* (1.01%), and uncultured *Veillonella* sp. (0.9%).

**Table 4:** ONT R10.4 output classification of the 4.5 kb *rrn* reads in characterizing the ZymoBIOMICS gut microbiome standard.

**Genus**	**Species**	**NCBI RRN**	**EMU**	**RDP**	**SILVA**	**Theoretical**
*Akkermansia*	*Akkermansia muciniphila*					0.97
*Bacteroides*	*Bacteroides fragilis*	15.55	15.53	15.77	4	9.94
*Bifidobacterium*	*Bifidobacterium adolescentis*	1.68	1.66	1.67	1.15	8.78
*Clostridioides*	*Clostridioides difficile*	4.52	4.38	4.41	4.13	2.62
*Clostridium*	*Clostridium perfringens*					<0.01
*Enterococcus*	*Enterococcus faecalis*					<0.01
*Escherichia*	*Escherichia coli*	8.12	8.08	8	3.56	12.12
*Faecalibacterium*	*Faecalibacterium prausnitzii*	9.43	9.32	2.28		17.63
*Faecalibacterium*	*uncultured Faecalibacterium sp*.			6.92		
*Fusobacterium*	*Fusobacterium nucleatum*	4.25	4.21	4.1	5.3	7.49
*Klebsiella*	*Klebsiella pneumoniae*				9.2	
*Limosilactobacillus*	*Limosilactobacillus fermentum*	1.03	1.01	1		9.63
*Methanobrevibacter*	*Methanobrevibacter smithii*					0.07
*Mycoplasma*	*Mycoplasma arthritidis*				0.51	
*Myroides*	*Myroides odoratimimus*				18.33	
*Prevotella*	*Prevotella corporis*	12.64	17.8	8.52	19.46	4.98
*Prevotella*	*Prevotella melaninogenica*	4.83				
*Prevotella*	*Prevotella sp. S4-BM14*			9.05		
*Roseburia*	*Roseburia hominis*	1.73	1.71	0.92	1.7	9.89
*Streptococcus*	*Streptococcus pneumoniae*				7.85	
*Veillonella*	*Veillonella rogosae*	0.69	6.79	3.72	3.18	15.87
*Veillonella*	*uncultured Veillonella sp*.			0.9		
*Veillonella*	*Veillonella atypica*	6.58				
*Veillonella*	*Veillonella dispar*	17.25	0.59	0.33		
*Veillonella*	*Veillonella parvula*	11.56	28.63	18.41	21.27	
*Veillonella*	*Veillonella sp. oral clone VeillD5*			1.01		
*Veillonella*	*Veillonella sp. SY-2*			11.33		
	*Salmonella enterica*					<0.01
	*uncultured bacterium adhufec225*			0.2		

Relative abundances are shown as the average across four replicates after applying sample abundance threshold filtering. Four databases were tested: NCBI RRN database (NCBI RRN), Ribosomal Database Project (RDP), the EMU database (EMU), and Silva-138 database (Silva-138). The values for the relative abundance of the Mock are obtained through the supplier's website and attached documents. Taxa absent using specific taxonomy databases are shown as missing values in their respective cells.

*Prevotella corporis*, which should have had an abundance of ~5%, was present at a higher abundance using any of the four databases (12.64, 8.52, 17.8, and 19.46%; NCBI RRN, RDP, and EMU, respectively). The RDP database classified 9.08% of the community as an undefined *Prevotella* sp. S4-BM14 while the NCBI RRN database uniquely identified 4.83% as *Prevotella melaninogenica*. The Silva-138 database performed better in this mock compared to the previous; however, incorrect taxa were still abundantly present. Silva-138 classified 18.33% of reads to *Myroides odaratimimus*, 0.51% of reads to *Mycoplasma arthritidis*, and 9.2% of reads to *Klebsiella pneumoniae* despite this taxon not being present in the sample. The RDP database produced the most “uncultured” taxa, even upon application of the relative abundance filter. As with the Zymo standard community, the relative abundances of the gut mock community were much closer to each replicate and database at the genus level than to the theoretical composition, but the abundances found were all within the documented deviation of 15% for each taxon in the community (Figure 6A).

### 3.7. RESCUE improves resolution accuracy and bridges database variability

**Figure 7:**
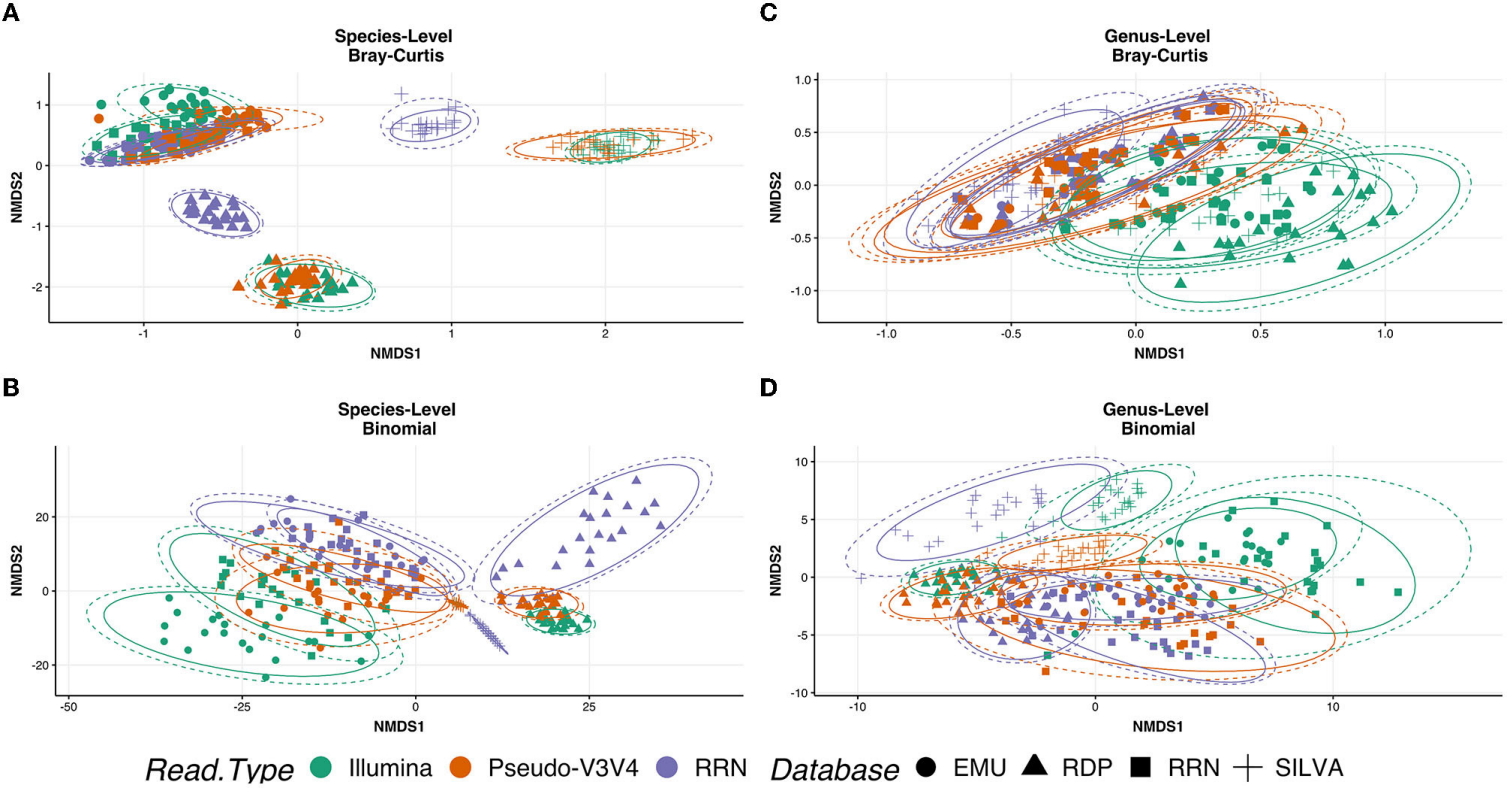
Ordination of the R10.4 Illumina comparison. NMDS ordination plots using “Bray” **(A, C)** and “binomial” **(C, D)**. Phyloseq object was stratified to species level **(A, B)** and genus level **(C, D)**. Calculations metrics from the “ordinate” function of the Phyloseq package. All samples were rarefied and kept in true “counts.” Sequence type variables RRN (purple), Pseudo-V3V4 (orange), and Illumina (green) are shown by the color of the data point. Database type: Emu (circle), RDP (triangle), RRN (square), and SILVA (cross) can be seen as the shape of each data point.

To determine whether RESCUE could be used in place of the standard Illumina sequencing, three read types were compared across four databases for the human saliva samples (*N* = 21 each): Illumina MiSeq V3V4 generated reads, 16S-ITS-23S ONT reads, and Pseudo-V3V4 reads subset from the ONT data. NMDS analysis at the species level, using Bray and binomial metrics, revealed a strong clustering of similarity by classification database, with additional sub-clusters between the sequence types (Figures 7A–D). At the species level, the Pseudo-V3V4 classifications clustered more closely to the Illumina V3V4 classifications within each respective database (Figures 7A, B, orange and purple). The *rrn* reads formed distinct clusters apart from the Pseudo-V3V4 reads and Illumina reads for both the RDP and Silva databases. This distinction can not only be best seen using Bray–Curtis metrics (Figure 7A) but can also be seen using binomial metrics, especially for RDP (Figure 7B).

At the genus level, the actual bacterial classification was shown to be similar across the three read types, allowing for NMDS separation to examine the bias of the sequencing platform and primers (Figures 7C, D). Even across databases, the *rrn* and Pseudo-V3V4 classifications are tightly clustered together at the genus level using Bray metrics (Figures 7C, D), with the classifications of Illumina reads through the NCBI RRN and EMU databases sharing some overlapping with the Nanopore-based data. Using the binomial metric at the genus level, which unweights high-abundance taxa, the read types are clustered by the database. Pseudo-V3V4 communities bridged the clusters formed between Illumina and *rrn* sequences, especially in the EMU and RRN databases.

**Figure 8:**
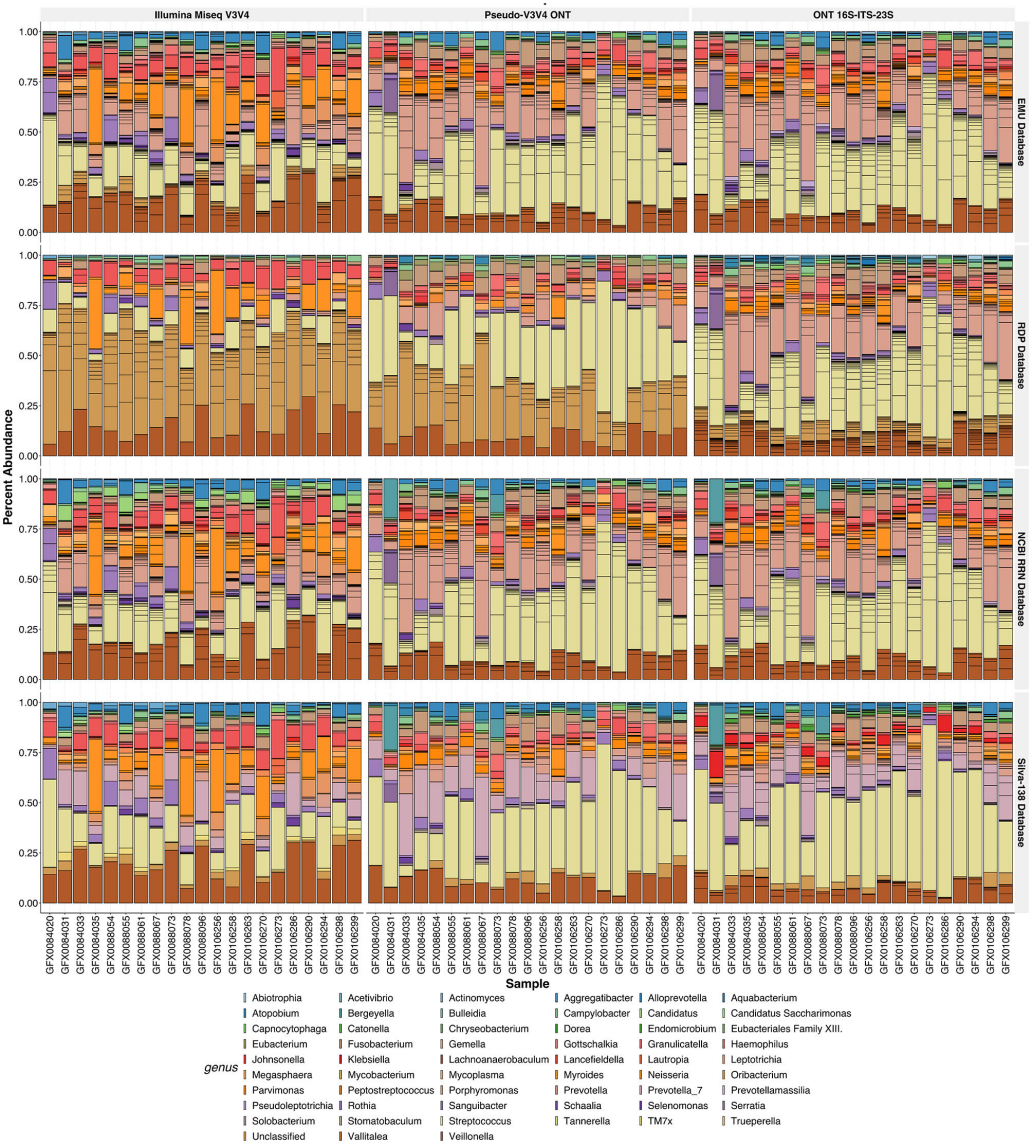
Relative abundance of the R10.4 Illumina comparison of human saliva. Relative abundances of the rarefied and filtered 21 samples were tested *via* three different sequence types (each vertical facet) across four different classification databases (each horizontal facet). The relative abundance of the species found is plotted, while the samples are colored by genus. Horizontal lines in each genus bar represent the abundance of species found within that genus.

The outlier behavior of the RDP and SILVA databases was assessed against human saliva samples (Figure 8). In this analysis, Pseudo-V3V4 and Illumina data were compared against the *rrn* reads across all four databases. A large share of the classification levels of the Illumina and Pseudo-V3V4 reads through the RDP database do not retain genus-level classifications and are listed as “Unclassified” (Figure 8). The short-read data for both the RDP and Silva database visibly stand out when compared against the *rrn* reads that appear very visually similar in classification across all four databases.

### 3.8. Partial 16s rRNA gene sequencing is ineffective at species and genus-level classification

The primary focus of the development of this pipeline that processes *rrn* reads for bacterial abundance analysis was to provide accurate resolution at both the genus and species levels of all taxa, including those that are difficult to classify. Using a traditional pipeline such as DADA2 and Silva v138 classification to process Illumina V3V4 data, the Silva v138 database does not normally allow for high-resolution classification in oral microbiome samples, partially due to many database entries not retaining species-level classification. Of the 50,280 reads obtained in a past publication (Ahrens et al., 2022) only approximately 95% of the reads are assigned taxonomy at the genus level and only 37% at the species-level classification. RDP, a more conservative and accurate database than SILVA or Greengenes (Edgar, 2018), in the RESCUE pipeline described here, is still able to classify 50% of reads at the genus-level classification for our Illumina dataset. Silva-138 with species-level taxonomy was also used in this benchmarking analysis to compare it to a traditional pipeline.

**Figure 9:**
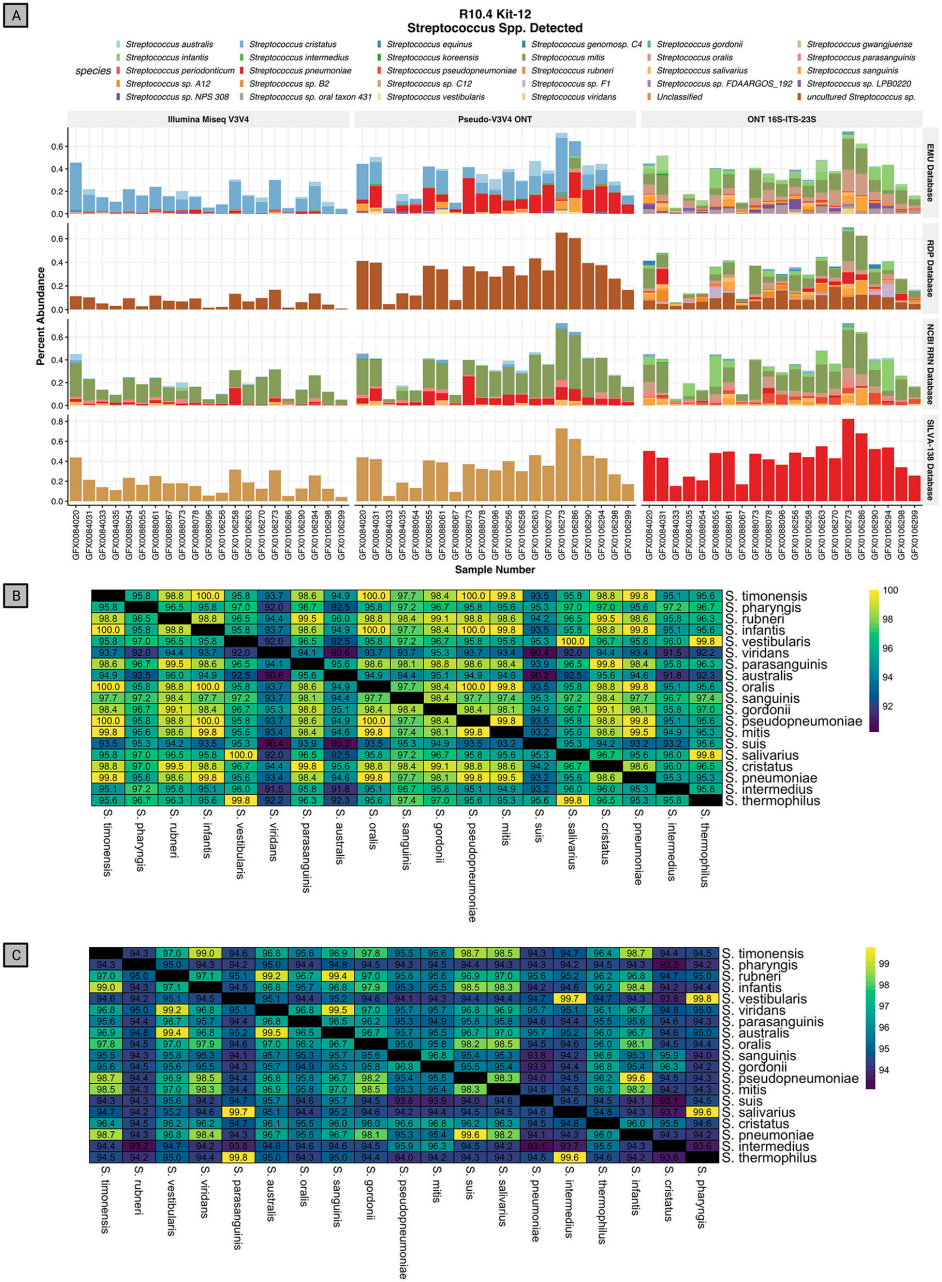
Relative abundance of *Streptococcus spp*. discovered in the human saliva data across three read types and four databases. **(A)** Relative abundance of species-level classification in the *Streptococcus* genus using RESCUE RRN Nanopore sequencing, Pseudo-V3V4 reads, and true Illumina reads using the EMU classifier across four databases. **(B)** *In silico* pairwise alignment of the 400 bp V3V4 region of 19 representative species in the genus *Streptococcus*. **(C)** *In silico* pairwise alignment of the 4.5 Kb RRN region of 19 representative species in the genus *Streptococcus*.

Using the *rrn* operon through the RESCUE pipeline shows the detection of 30 species within the *Streptococcus* genus (Figure 9A). For short-read V3V4 regions, using either Illumina or Psuedo-V3V4 through Nanopore, produces a consensus among species classifications for each database that differs from the full-length *rrn* reads. The short-read classifications even differ greatly among the four classification databases. For short reads at the species level, the EMU database classifies primarily *S. cristatus* and *S. pneumoniae*, the RDP and Silva databases are entirely “uncultured *Streptococcus* spp.” or “Unclassified,” and the NCBI RRN database is primarily *S. mitis and S. pneumoniae*. While all four databases differ for the V3V4 classification of *Streptococcus*, the long-read RESCUE (*rrn*) classifications all appear to converge among the primary community of *S. mitis, S. oralis, S. sanguinis*, and *S. infantis*. Running the *rrn* reads through the Silva-138 database allowed for species-level classification, but interestingly all *Streptococcus* species were classified as *Streptococcus pneumonia* similar to the behavior in the mock community.

Short-read V3V4 sequencing is ineffective for the classification of *Streptococcus*, a major taxon in saliva that is difficult to classify at the species level. *In silico* analysis shows how the 400 bp V3V4 region is unable to provide species-level resolution by aligning the V3V4 portion of 19 representative *Streptococcus* species found in rrn sequencing (Figure 9B). Across the pairwise alignment of these V3V4 regions, several instances arise where two distinct species have 100% identical V3V4 hypervariable regions, such as *Streptococcus mitis* and *Streptococcus infantis*. Most of the remaining comparisons differ by one or two single nucleotide polymorphisms (SNPs). Using just the V3V4 region for *Streptococcus* spp. classification is challenging without the greater context from the entire *rrn* operon.

When performing pairwise alignments of the *rrn* of the same representative species, each species has a minimum of nine distinct SNP mutations in the 4.5Kb *rrn* fragment produced by the *rrn* primers (Figure 9C). The same example of *S. mitis* and *S. infantis* described above, now only have 95.7% identity between each other. This leaves enough context of the *rrn* operon, with >99% raw read accuracy, to accurately classify the species level of *Streptococcus*. Numerous other examples have been studied, such as *Akkermansia, Enterobacter, Enterococcus, Klebsiella, Listeria, Staphylococcus*, and *Prevotella*, which all have poor identification even at the genus level when using the Silva database for V3V4 classification (Abellan-Schneyder et al., 2021).

## 4. Discussion

Here, we created and validated a pipeline to sequence, classify, and analyze samples for their bacterial compositions using 16S-ITS-23S rRNA amplicons produced by Nanopore sequencing. As a few laboratories have recently shepherded the turn toward full-length 16S and 16S-ITS-23S rRNA (Curry et al., 2021; Graf et al., 2021; Kinoshita et al., 2021; Seol et al., 2022), we sought to assist in this transition by finding any limitations of moving away from Illumina and testing the generalizability of Illumina-based results to *rrn* based. We combined the best aspects of multiple validated, third-party, and open-sourced, Nanopore-based programs into one publicly available workflow. Curry et al. (2021) developed the EMU classifier that is validated as the best performer on Nanopore error profiles. Kinoshita et al. (2021) curated a custom *rrn* database of 16S-23S rDNA from RefSeq-verified genomes (Kinoshita et al., 2021). Martijn et al. (2019) designed a primer that would cover 98.9% of bacterial 23S (U2428R). RESCUE wraps all these approaches and software into a one-line script, helping to bridge access to programs and broaden the use of 4.5 kb rrn amplicons for taxonomic classification.

In testing the initial R9.4 Kit 10 chemistry commonly used for genome assemblies, accurate species-level classification was obtained of the Zymo mock community at an average quality score of the final reads at Q13.3 or ~95.3% read accuracy (Table 1). Although *rrn* sequencing with the R9.4 flow cells displayed enough accuracy for species-level classification of most taxa, the accuracy of using R10.4 flow cells or better in the analysis will increase the confidence in the findings. Using the R10.4 flow cell and Kit12 chemistry, final reads inputted into the EMU classifier at greater than Q20 median accuracy were observed (Table 2), which corresponds to 99.0% read accuracy with mostly species-level classification in the microbial mock community. In testing the gut microbiome mock samples, a lower limit of detection was found, which caused a failure to detect four taxa, each at <1% relative abundance. While this could be a true lower threshold of detection of the pipeline, despite 1% abundance being at least 20 reads, this was not the result of primer bias as *Salmonella enterica* was correctly detected in the other mock samples. The raw read accuracy of ONT chemistry and base callers continue to improve, as can be seen from the jump in quality just between Guppy v5.1.13 and v6.1.7, and the updated ONT release with Kit14 chemistry. This increases the throughput of high-quality reads going into the classifier which increases the depth of detection and resolution of classification.

To account for the 73 reads identified in the single null control used in the R10.4 runs, thresholding was increased from 0.0056 to 0.0073% from the R9.4 to the R10.4 runs. RESCUE users should always include a true null barcode combination of the 96 provided in multiplexed amplicon libraries to determine the level of barcode thresholding due to demultiplexing errors. The demultiplexing incorporated into the RESCUE pipeline is stringent, as all 16 bases of both barcodes must be found with 100% accuracy, in the correct orientation, and fit under the global length and Q10 constraints. However, the Phred scores of each barcode nucleotide are remarkably high on average, often over Q30. In the RESCUE pipeline options, the implementation of the “-b” setting allows for multiple mismatches if users choose not to use the zero-mismatch option. Theoretically, the primers retain 7 substitution differences from one another in a global alignment. Increasing mismatch allowance appears to retain higher amounts of reads, as changing mismatch allowance to 1, 2, and 3 errors per barcode, allow for the retention of 43, 46, and 51% of reads, respectively (Supplementary Table 1). There appears to be an ideal spot of around 1 mismatch, as 2 or more begin raising the number of reads found in the null control sample. This in turn should increase the downstream abundance filtering to remove this effect.

When comparing the classification of the MiSeq-generated V3V4 fastq files alongside 4.5 Kb *rrn* reads and pseudo-V3V4 data of the same DNA samples, the influence on read length can be more accurately determined against the noise of primer and platform biases. The distance matrices at the species level reliably show how classifications from full-length 16S rRNA gene databases (EMU, NCBI RRN, and Silva-138) benefit from longer reads. The genus-level Bray ordination is more revealing of the inherent differences in the RESCUE approach vs. Illumina MiSeq which undoubtedly misses some taxon. The weighted Bray index reveals how Illumina reads show cluster separation from both Nanopore read types. These differences hold true between classification databases that can be attributed to low abundance noise.

After looking at the classification results, short-read V3V4 sequencing makes it exceedingly difficult to classify reads assigned to specific genera like *Streptococcus*. The classifier embedded in RESCUE obtains species-level results, if available, while traditional pipelines like DADA2 do not by default. This highlights the ability of 4.5kb *rrn* reads to obtain an accurate consensus of the community composition vs. the short-read V3V4 reads, sequenced through Illumina or Pseudo-V3V4 Nanopore. Multiple databases are used across 16S rRNA community analysis research, and as shown here the choice of database impacts the resulting classification of short-read V3V4 sequencing. Using the long-read *rrn* data, the classification is more uniform and converges across all databases at both species and genus-level. It is important to note that the EMU classifier and by default RESCUE utilize longest read scoring, meaning that the longer alignment wins even if this alignment is only in a certain region of the read. This may explain why the Zymo mock and saliva data were skewed with *Streptococcus pneumoniae*.

Some limitations are inevitable as found in our RESCUE pipeline. Prior *in silico* classification of available genomes determined that certain symbiotic and slow-growing taxa in soil have unlinked *rrn* operons, where the distances between the 16S and 23S rRNA genes are >1,500 bp (Brewer et al., 2020). In this study, increasing extension time failed to capture more diversity, suggesting that unlinked *rrn* operons are rare in the samples used here. The largest ITS we found was from *Candidatus Saccharibacteria*, with an average total amplicon size of 5,700 bp. Further testing needs to be done to determine if the high molecular weight (HMW) smearing on the gel was the incomplete amplification of unlinked *rrn* operon genomes. One could argue, however, that the benefit of the increased size of *rrn* sequencing over full-length 16S outweighs the omission of unlinked *rrn* operons, especially if the sample type is void of these taxa such as human samples (Kinoshita et al., 2021). Admittedly, some labs may still choose to use full-length 16S primers due to their reliability, especially in unknown samples where unlinked *rrn* taxa may be present.

One commonly cited barrier to the adoption of Nanopore sequencing for full-length 16S rRNA genes or larger fragments is the perceived high error rate of the platform. However, the ONT platform continues to improve, leading to a significant reduction in error rates. As Nanopore sequencing technology continues to advance, the challenges associated with reduced throughput of clean data, low-prevalence noise taxa in mock samples, and barcode crosstalk can be mitigated effectively or even resolved. Our analysis demonstrates that the benefits of increased confidence across databases, enhanced taxonomic resolution down to species level, and reduced noise compared to Illumina-based approaches arguably outweigh the downsides. Our findings show that the ONT long-read chemistry is now surpassing an average of 99.0% raw read accuracy in processed reads. Additionally, the RESCUE pipeline and taxonomic analysis using long-read Nanopore contribute to the improved resolution required for many bacterial diversity analyses. While the current accuracy of 99% achieved by ONT is still notably lower than Illumina sequencing and translates to 45 errors per RESCUE read, the longer read length achievable by ONT provides sufficient context to achieve species-level classification despite this error rate. Furthermore, our analysis indicates that the error distribution in reads from known mocks is less concentrated in the middle, implying enhanced resolution in most hypervariable regions. While ONT and RESCUE may not yet be suitable for single nucleotide level analysis, the available evidence indicates that the current error rate and RESCUE parameters enable the general classification of samples and provide enhanced species-level resolution.

## Data availability statement

The datasets presented in this study can be found in online repositories. The names of the repository/repositories and accession number(s) can be found below: PRJNA982569 (SRA). All code and instructions for RESCUE can be found at https://github.com/josephpetrone/RESCUE.

## Ethics statement

For the human saliva used to generate previous Illumina data and current RESCUE data, the study was approved by and carried out in accordance with the University of Florida Institutional Review Board (IRB), with study approval IRB study #201801744. All methods were carried out in accordance with relevant guidelines and regulations.

## Author contributions

JP conceptualized the pipeline, wrote the main coding portions of the pipeline, and formulated the first draft of the manuscript. PR and CG provided valuable benchwork, conceptualization, and interpretation of the data. PM contributed to the RESCUE code and R scripts. AA contributed to the saliva data and helped investigate the findings. LR and ET provided guidance, funding, and conceptualization. All authors listed contributed to the discussions of the study and the synthesis and editing of the publication, contributed to the article, and approved the submitted version.
