# Supplementary material for: RESCUE: a validated Nanopore pipeline to classify bacteria through long-read, 16S-ITS-23S rRNA sequencing
Source: Front Microbiol. 2023 Jul 20;14:1201064. doi: 10.3389/fmicb.2023.1201064 (PMC10402275; doi:10.3389/fmicb.2023.1201064)
Supplement: Supplementary file 1 [file Data_Sheet_1.docx]

Supplementary Material

RESCUE: a Validated Nanopore Pipeline to Classify Bacteria Through Long-Read, 16S-ITS-23S rRNA Sequencing

**Joseph R. Petrone, Paula Rios Glusberger, Christian D. George, Patricia L. Milletich, Angelica P. Ahrens, Luiz F.W. Roesch, and Eric W. Triplett^*^**

*** Correspondence:** Eric W. Triplett: ewt@ufl.edu

# 1 Supplementary Figures and Tables

**Supplementary Table 1. ONT R10.4 Run-4 Demultiplexing output of the 4.5kb *rrn* reads based on barcode mismatch threshold.** The first column denotes the mismatch allowance for each barcode on the initial demultiplexing. The second column denotes the number of reads taken forward, followed by the reads found in the null negative control, the percentage of null reads to total, and the percentage kept from the filtering step, followed by traditional NanoStat statistics.

| Mismatches Allowed | Reads  (•10^6^) | Reads  In Null | %  Total | %  Kept | Bases  (Gb) | Avg  Len | Med  Len | Avg  Q | Med  Q | %  ≥Q12 | %  ≥Q15 |
| --- | --- | --- | --- | --- | --- | --- | --- | --- | --- | --- | --- |
| 0 - Mismatch | 1.00 | 73 | .0073 | 39 | 4.64 | 4629 | 4534 | 18.6 | 18.9 | 98 | 87 |
| 1 - Mismatch | 1.11 | 79 | .0071 | 43 | 5.13 | 4628 | 4534 | 18.4 | 18.8 | 97 | 85 |
| 2 - Mismatch | 1.19 | 105 | .0088 | 46 | 5.49 | 4627 | 4534 | 18.3 | 18.7 | 96 | 84 |
| 3 - Mismatch | 1.33 | 390 | .0294 | 51 | 6.13 | 4617 | 4523 | 18.0 | 18.4 | 95 | 81 |


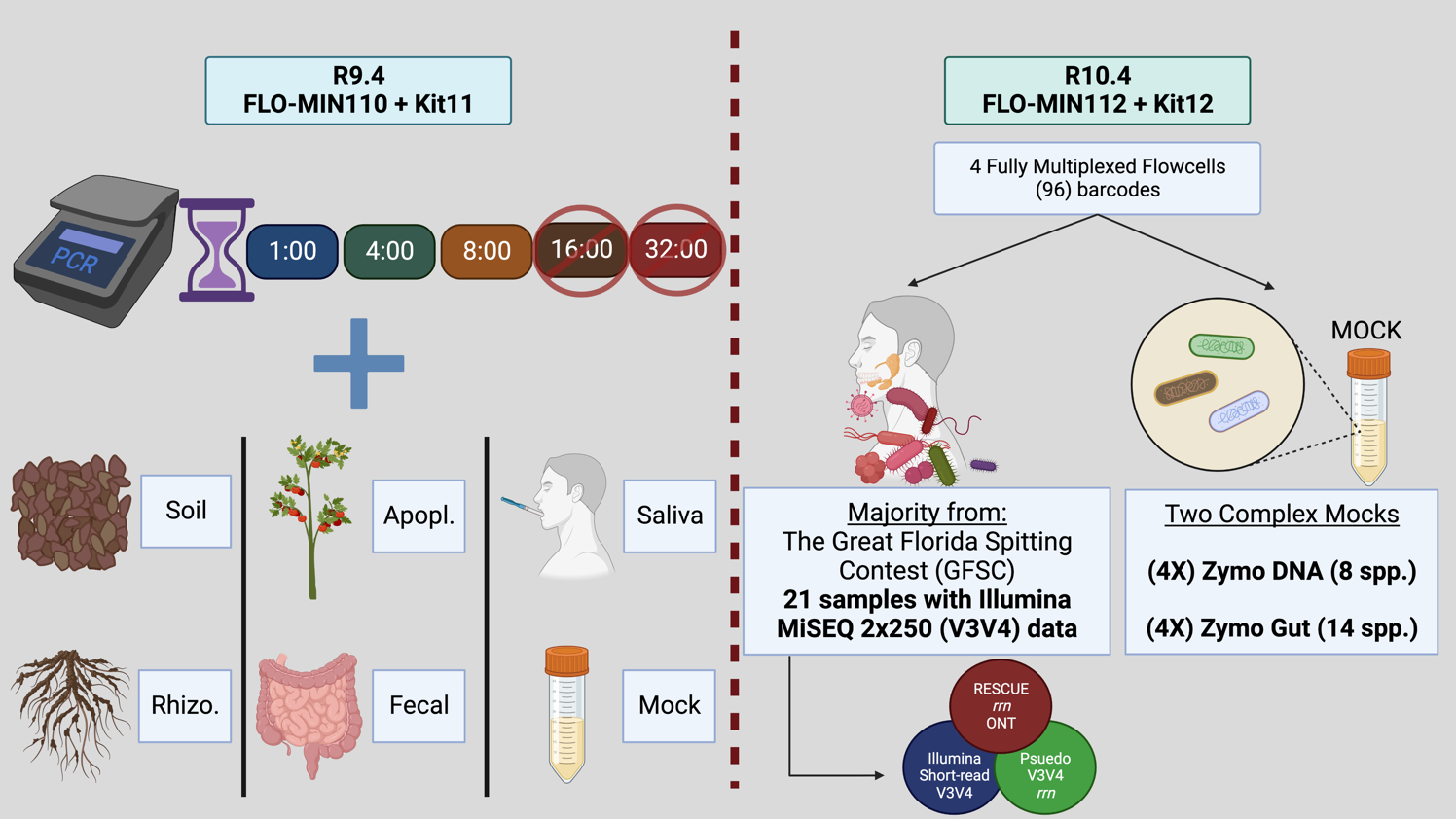


**Supplementary Figure 1**. **Graphical abstract of the RESCUE pipeline benchmarking experiments**. (Left) The R9.4 ONT flowcell and Kit11 consisted of testing 5 polymerase extension times in the PCR reaction across 6 sample types: bulk soil, rhizosphere-associated soil, tomato apoplastic fluid, human feces, human saliva, and Zymo mock communities. (Right) The R10.4 Kit12 trial consisted of 4 fully multiplexed flowcells consisting of 96 samples per flowcell. Within that multiplex, 21 samples were loaded onto the runs that we had previously sequenced using Illumina MiSeq V3V4 primers with 2x250 chemistry. The RESCUE *rrn* output of the DNA for those previously sequenced samples was compared to their Illumina data output and a pseudo-V3V4 subset of the RESCUE reads was done by clipping the reads to the hypothetical length of the Illumina data. Additionally, two mock communities. The Zymo Microbial DNA and Zymo Gut Microbiome Standard were included on each R10.4 run.


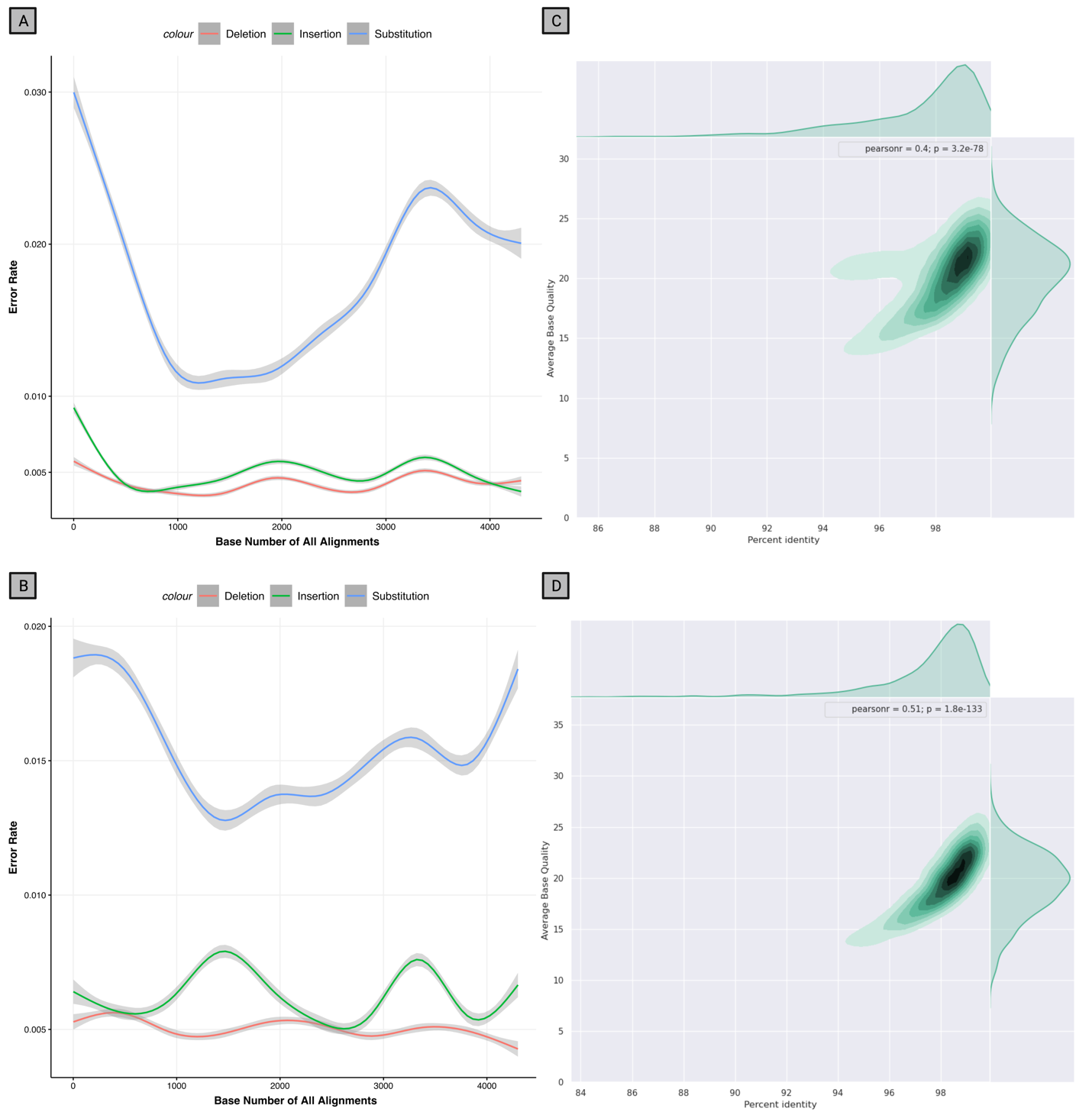


**Supplementary Figure 2.** **True error rates of the RESCUE *rrn* reads classified to the Zymo Microbial Mock and Zymo Gut Microbiome Standard communities**. (A) The raw mapping files of all 4 Zymo microbial mock DNA communities against their hypothetical genomes pulled from the supplier website. The X-axis shows the base number of the entire alignment while the y-axis shows the error rate out of 1.0. The type of errors can be shown as substitution (blue), insertion (green), and deletion (red). (C) The NanoPlot heat plot of the mapping files of all 4 Zymo microbial mock DNA communities against their hypothetical genomes pulled from the supplier website. The X-axis shows the percent identity of the alignments while the y-axis shows the average base quality. The darkness of the dot plot shows density while distributions can be seen on the extremes of each axis. (B) The raw mapping files of all 4 Zymo Microbiome Standard DNA communities against their hypothetical genomes pulled from the supplier website. The X-axis shows the base number of the entire alignment while the y-axis shows the error rate out of 1.0. The type of errors can be shown as substitution (blue), insertion (green), and deletion (red). (D) The NanoPlot heat plot of the mapping files of all 4 Zymo Microbiome Standard DNA communities against their hypothetical genomes pulled from the supplier website. The X-axis shows the percent identity of the alignments while the y-axis shows the average base quality. The darkness of the dot plot shows density while distributions can be seen on the extremes of each axis.


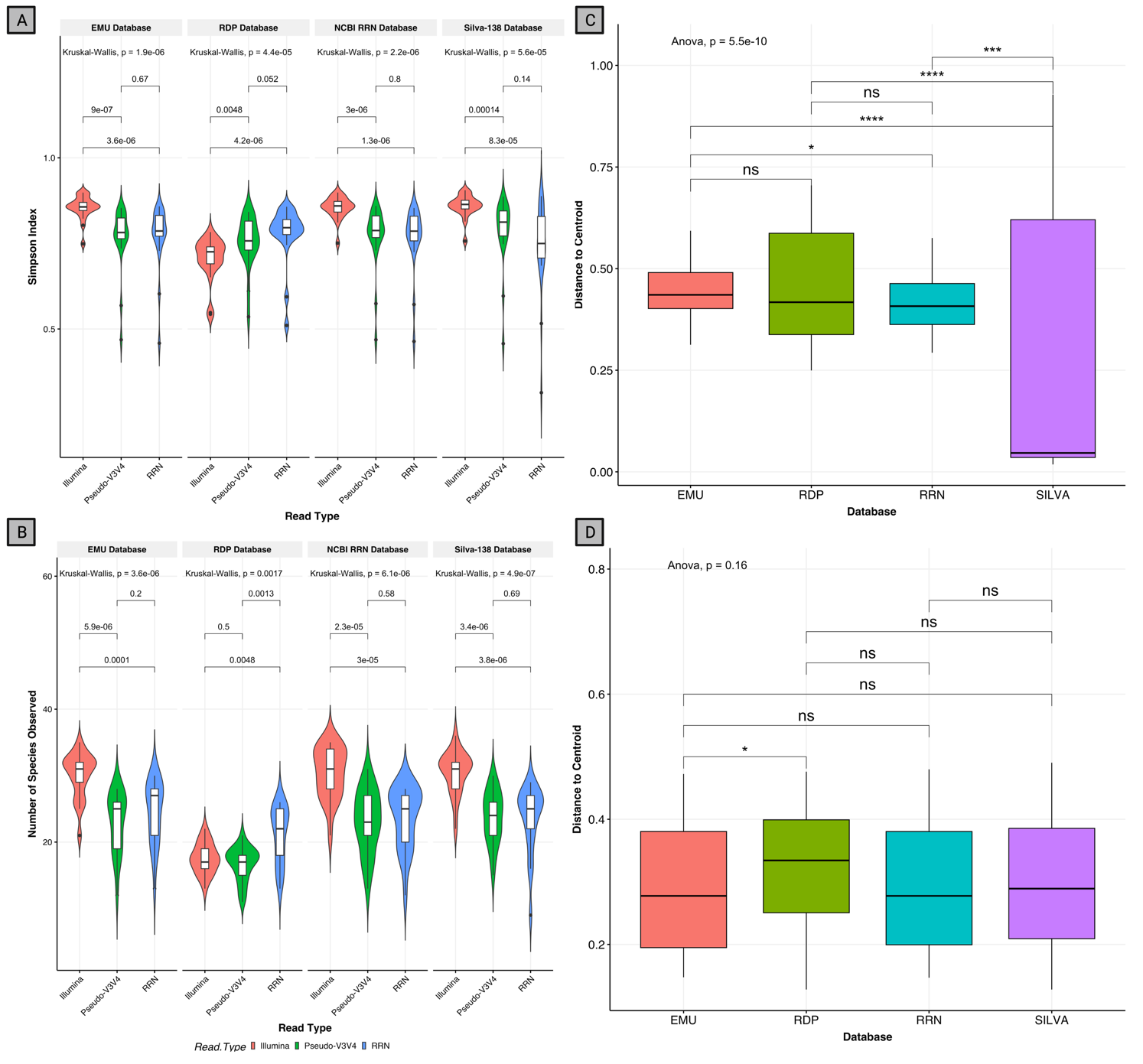


**Supplementary Figure 3.** **Alpha and beta-diversity measures of the R10.4 rrn Illumina comparison further stratified**. **(A)** Alpha diversity by the measure of Simpson Evenness at genus-level and **(B)** Observed richness at genus-level. The database is faceted on top of each read grouping while the sequence type can be seen as red (Illumina), green (Pseudo-V3V4), and blue (RRN). Kruskal-Wallis and pairwise chi-squared were done and p.adj is shown. **(C)** Beta-dispersion metrics of distance to the centroid. Sprawl between sample centers can be seen on the y-axis while each sequence database is plotted separately on the x-axis. Non-parametric ANOVA and pairwise t-test are shown here at the genus-level. **(D)** Beta-dispersion metrics of distance to the centroid. Sprawl between sample centers can be seen on the y-axis while each database type is plotted separately on the x-axis. Non-parametric ANOVA and pairwise t-test are shown here at the species-level.
